# Inhibitory Control Predicts Growth in Irregular Word Reading: Evidence From a Large-Scale Longitudinal Study

**DOI:** 10.1037/dev0001563

**Published:** 2023-08-31

**Authors:** Yani Qiu, Sarah Griffiths, Courtenay Norbury, J. S. H. Taylor

**Affiliations:** 1 Division of Psychology and Language Sciences, University College London; 2 Department of Special Needs Education, University of Oslo

**Keywords:** irregular word reading, inhibitory control, reading development, decoding, vocabulary

## Abstract

Irregular words cannot be read correctly by decoding letters into sounds using the most common letter–sound mapping relations. They are difficult to read and learn. Cognitive models of word reading and development as well as empirical data suggest that inhibitory control might be important for irregular word reading and its development. The current study tested this in a U.K. population-based cohort (*N* = 529, 52.74% male, 90.17% White) in which children were assessed longitudinally at ages 5–6, 7–8, and 10–11 years. Results showed that inhibitory control did not predict concurrent irregular word reading after controlling for the covariates of decoding and vocabulary. However, inhibitory control made a small but significant contribution to growth in irregular word reading across time points, over and above vocabulary (decoding did not predict growth). Therefore, children might need to inhibit the predisposition to overgeneralize the most common relations between letters and sounds when learning to read irregular words.

## Public Significance Statement

Our study suggests that children might need to inhibit the tendency to use the most common letter–sound mapping relations in order to develop their ability to read irregular words. Teachers may want to encourage students to think whether the pronunciation they generate for a written word matches a spoken word they know, and whether the spelling they generate for an oral vocabulary item matches the written form in print.

Reading aloud involves sounding out words from their printed form. In alphabetic languages, regular words can be read correctly using knowledge of the most common relations between letters and sounds (grapheme–phoneme correspondences [GPCs]; e.g., letters “ea” are pronounced as /i:/ in “freak,” “creak,” “leak” in English; Coltheart et al., 1993). However, irregular words contain some letters that contravene GPCs (e.g., “break,” in which “ea” is pronounced as /eɪ/). Many writing systems are not perfectly regular (Dehaene, 2010); for example, in English, letters are not pronounced in accordance with GPCs approximately 30% of the time in children’s printed word corpora (Caravolas et al., 2012). Irregular words are more difficult to read and learn than regular words (e.g., Castles et al., 2009), prompting questions as to what cognitive processes underlie successful irregular word reading and its development.

The following sections will first focus on irregular word reading, and then on the development of irregular word reading. Each section outlines previous research which suggests that vocabulary (knowledge of known words) and decoding (the ability to use GPCs to decode letters into sounds; Gough & Tunmer, 1986) are important factors in reading and learning irregular words, and then presents evidence that inhibitory control might make unique contributions. Inhibitory control is the ability to override a strong predisposition and develop new ways of behaving to “do what’s more appropriate” (Diamond, 2013, p. 137; Norman & Shallice, 1986). It is supported by brain networks that maintain attention, monitor performance, initiate control, and adjust behavior (Dosenbach et al., 2007; Stuss & Alexander, 2007).

## Irregular Word Reading

### Decoding and Vocabulary in Irregular Word Reading

**Figure 1 fig1:**
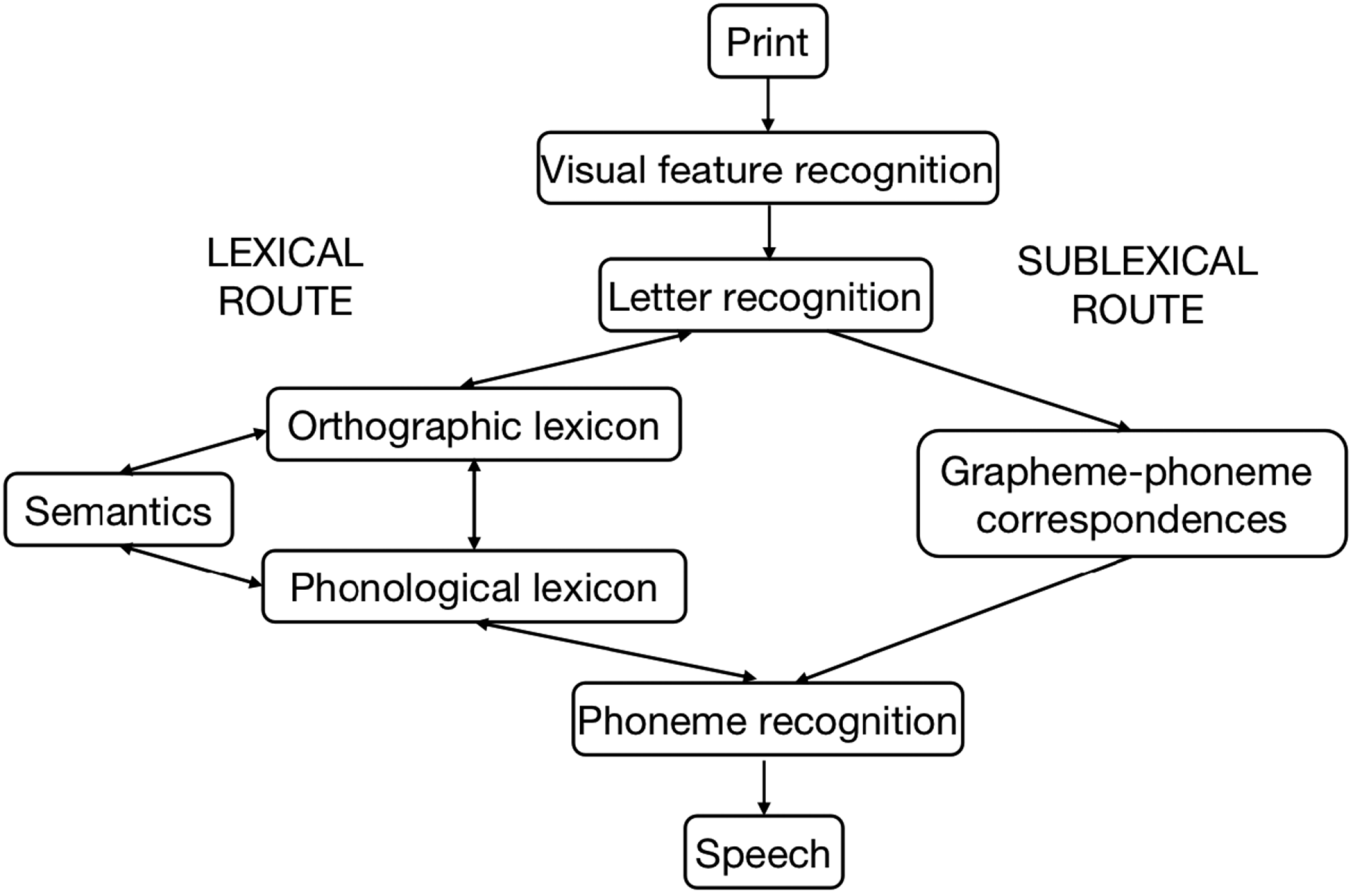
The Dual-Route Cascaded Model *Note*. Adapted from “DRC: A Dual Route Cascaded Model of Visual Word Recognition and Reading Aloud,” by M. Coltheart, K. Rastle, C. Perry, R. Langdon, and J. Ziegler, 2001, *Psychological Review, 108*(1), p. 214 (https://doi.org/10.1037/0033-295X.108.1.204). Copyright 2001 by the American Psychological Association.

Decoding and vocabulary significantly predict concurrent irregular word reading (Johnston et al., 2014; Ricketts et al., 2007; Steacy et al., 2017). This accords with the dual-route cascaded (DRC) model (Coltheart et al., 2001; Figure 1), which reads written words via two routes in parallel. The sublexical route embodies processes underlying decoding. It uses GPCs to decode graphemes (e.g., B, R, EA, K) of written words (e.g., “break”) into phonemes (/bri:k/). The other route is the lexical route, which accesses vocabulary knowledge. It maps letters onto a whole-word written representation in the orthographic lexicon (BREAK), which then links to a corresponding pronunciation in the phonological lexicon (/breɪk/), with or without semantic input. The phoneme output from both routes converge at the phoneme recognition node. For irregular words, the decoded output (/bri:k/) and the lexical output (/breɪk/) match on some phoneme slots (/b/, /r/, /k/), which helps the word to reach the reading aloud threshold. Therefore, the DRC model predicts that both decoding and vocabulary should contribute to concurrent irregular word reading.[Fig fig1]

### Inhibitory Control in Irregular Word Reading

When the DRC model (Coltheart et al., 2001; Figure 1) reads irregular words (e.g., “break”), the decoded output (/bri:k/) and the lexical output (/breɪk/) conflict with each other at certain phoneme slots (e.g., the vowel, /i:/ or /eɪ/). To resolve this conflict, a large value was set for the *Phoneme to phoneme inhibition* parameter at the phoneme recognition node. This reduces the activation level at the phoneme slots where the decoded and lexical output disagree, and allows more processing cycles to determine the phoneme (e.g., /i:/ or /eɪ/; will settle on the lexical phoneme /eɪ/ due to its stronger activation than the decoded one). Therefore, the DRC model proposes the following cognitive processes might be employed when we read irregular words: inhibiting from reading aloud straight away, allowing time to settle on an output, and finally reading aloud the lexical output.

Analysis of error patterns in human readers revealed that the majority of errors in reading irregular words were the regularized decoded output (e.g., reading “break” as /bri:k/; Treiman et al., 1995, Experiments 3 and 4, in which readers read known words). This indicates that readers have the predisposition to read aloud the decoded output, even when the lexical output is also available. These data therefore also suggest that to correctly read irregular words, human readers need to inhibit the predisposition to read aloud the decoded output without considering the lexical output. Any effect of inhibitory control on irregular word reading should only be observable once readers have learned GPCs in addition to some irregular words, as only then will the predisposition to read aloud the decoded output be established and the lexical output be available.

## Development of Irregular Word Reading

### Decoding and Vocabulary in the Development of Irregular Word Reading

**Figure 2 fig2:**
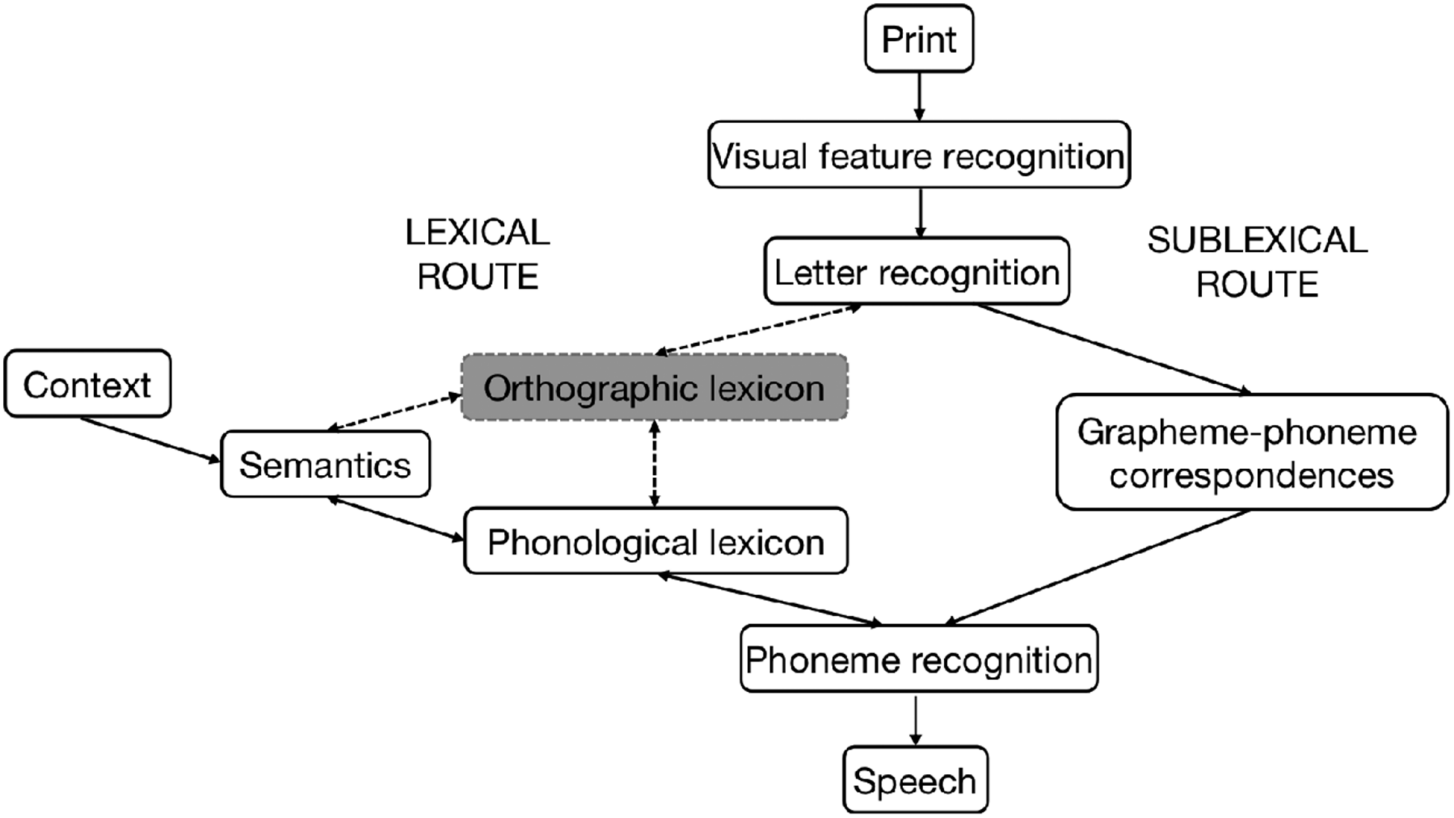
The Self-Teaching Dual-Route Cascaded Model *Note*. To simulate that the model has not learned the novel written word, the orthographic lexicon is grayed out, lines between the orthographic lexicon, phonological lexicon, semantics, and letter recognition are disconnected. Once learned, the orthographic lexicon is ungrayed, lines become connected, so that the lexical route is fully established. From “A Computational Model of the Self-Teaching Hypothesis Based on the Dual-Route Cascaded Model of Reading,” by S. C. Pritchard, M. Coltheart, E. Marinus, and A. Castles, 2018, *Cognitive Science*, *42*(3), 722–770 (https://doi.org/10.1111/cogs.12571). Copyright 2018 by Wiley. Adapted with permission.

Longitudinal behavioral data show that decoding and vocabulary significantly predict irregular word reading later in development (Nation & Snowling, 2004; Ricketts et al., 2007). This is consistent with a self-teaching version of the DRC (ST-DRC) model (Pritchard et al., 2018; Figure 2; for the self-teaching hypothesis, see Share, 1995). When the ST-DRC model encounters a novel written word such as “chef,” the lexical route is not fully established, and the model cannot read the word along this route. However, decoding provides an opportunity to self-teach the word. The model uses GPCs to decode graphemes into phonemes via the sublexical route, generating /tʃef/ (“ch” being pronounced as in “church”). Additionally, contextual information is usually available (although ambiguous), and this activates relevant concepts in the semantics node (e.g., the concepts of a person who works in a restaurant), which in turn activates the pronunciation of corresponding known spoken words in the phonological lexicon (/weɪtə/, /weɪtrəs/, /ʃef/ for words “waiter,” “waitress,” and “chef,” respectively; “ch” in “chef” being pronounced as “sh” as in “shake”).[Fig fig2]

The decoded output (/tʃef/) and the phonological lexical output (/ʃef/) match on some phoneme slots (/e/, /f/). For these phoneme slots, both decoding and oral vocabulary contribute activation to help reach the *SpokenWordRecognisedThreshold*, which allows the novel written word (“chef”) to be recognized as a known spoken word (/ʃef/). Once the known spoken word is recognized (/ʃef/), learning is initiated. The whole-word orthographic representation (CHEF) becomes established in the orthographic lexicon. It becomes connected with the whole-word phonology (/ʃef/), letters (“chef”), and semantics. A full lexical route is then developed for the word “chef.” Next time when the model encounters the word, it generates output via both the lexical route and the sublexical route, as in the DRC model.

### Inhibitory Control in the Development of Irregular Word Reading

When the ST-DRC model (Pritchard et al., 2018; Figure 2) self-teaches irregular words (e.g., “chef”), the decoded output (/tʃef/, “ch” being pronounced as in “church”) conflicts with the plausible lexical candidate (/ʃef/, “ch” being pronounced as “sh” as in “shake”) at certain phoneme slots (/tʃ/ or /ʃ/). To resolve this conflict, a large value was set for the *PhonemePhonlexInhibition* parameter at the phoneme recognition node. This parameter reduces activation at the phoneme slots where output disagrees (e.g., /tʃ/ or /ʃ/), and prevents the model from using the decoded /tʃef/ to develop the lexical route (i.e., connecting the /tʃef/ to the orthographic representation CHEF in the orthographic lexicon, which then connects to semantics and letters). Instead, the inhibition parameter allows more processing cycles to recognize the correct phonological lexical output /ʃef/, connect it to the orthographic lexical representation, and develop the full lexical route. Therefore, the ST-DRC model suggests that human readers might need to inhibit themselves from using the decoded output to develop the lexical route, and instead allow time to recognize the correct phonological lexical output and use that to develop the correct lexical route. This is supported by empirical data. Analysis of children’s self-teaching outcomes showed that 55% of irregular words were pronounced using the decoded output (Murray et al., 2022), indicating children’s predisposition to overuse the decoded output when self-teaching irregular words. To correctly self-teach irregular words, children might need to inhibit this predisposition.

The ST-DRC model assumes that, after the phonological lexical output is recognized (e.g., /ʃef/), it can be smoothly connected to the correct orthographic representation (CHEF), which will be stored in the orthographic lexicon. However, empirical studies show that developing the correct orthographic representation and connections is not easy for irregular words (e.g., Wang et al., 2011, 2012). Wang et al. (2011; Experiment 2) examined 7- to 9-year-old children’s self-teaching of novel written irregular words. First, children were familiarized with oral vocabulary knowledge (semantics and phonological lexicon) of the words. They were then instructed to use this knowledge to assist reading aloud of these words, either in contextually rich stories (context condition) or lists (no-context condition). Their orthographic representations of these words were tested immediately and after a 10-day delay. Results showed that, across conditions (context, no-context; immediate, delay) and orthographic tests (spelling, orthographic choice, orthographic decision), around 50% of words were self-taught with a regularized orthographic representation (e.g., SHEF). This reflects children’s robust predisposition to overgeneralize GPCs when developing orthographic representations for irregular words. Therefore, this predisposition might need to be inhibited before a stable orthographic representation (e.g., CHEF) can be established in the orthographic lexicon and the lexical route can be correctly developed.

## The Current Study

The evidence so far suggests that inhibitory control might be important for irregular word reading and its development. However, this has not been tested. The current study aimed to fill in this gap. We hypothesized that (a) inhibitory control would correlate with concurrent irregular word reading; (b) inhibitory control would predict concurrent irregular word reading after controlling for decoding and vocabulary, but only at later years when the sublexical and lexical routes have developed to a certain extent; and (c) inhibitory control would predict growth in irregular word reading, after controlling for decoding and vocabulary. We also planned to test whether and to what extent decoding and vocabulary predict growth in irregular word reading. To test these hypotheses, a secondary data analysis was conducted on data collected at three time points in the Surrey Communication and Language in Education Study (SCALES), a longitudinal U.K. population-based study that tracked children’s language, academic, and cognitive development (Norbury, 2022).

## Method

### Participants

Participant recruitment and selection procedures are described in detail in Norbury et al. (2016). The SCALES screened 7,267 participants from 161 primary schools in Surrey, England for language difficulties at school entry using the teacher-rated version of the Children’s Communication Checklist (CCC-S; from Children’s Communication Checklist 2, Bishop, 2003). Based on sex-and-age-specific cutoff scores on the CCC-S (one standard deviation above the means), stratified random sampling was performed and 636 monolingual English-speaking participants were selected. Participants with CCC-S score higher than the cutoff points (more language difficulties) were oversampled. The sampling procedures provided diversity in language abilities of participants, which benefits the current study as it ensures sufficient variation in language measures of interest (irregular word reading, decoding, vocabulary).

Among the selected sample, 529 participants participated in an in-depth assessment (90.17% White, 2.27% Asian, 0.38% Black, 4.54% Mixed, 2.65% Other). They were then assessed longitudinally. The current study used data collected in Year 1 (2012–2013; 529 children, 279 male; *M*_age_: 5.97 years, range: 5.08–6.83), Year 3 (2014–2015; 503 children, 263 male; *M*_age_: 7.94 years, range: 7.08–9.25), and Year 6 (2017–2018; 384 children, 196 male; *M*_age_: 11.16 years, range: 10.42–12.00). As in the preregistration (https://osf.io/vah82), we used Monte Carlo simulations (Muthén & Muthén, 2002; 10,000 iterations) to determine the statistical power available to detect minimal effects of interest (*r* = .196; Open Science Collaboration, 2015) for hypothesis (c), the most complicated and sample-size-demanding analysis in the current study. Results showed more than 98% power to detect effects of interest, given 529 participants and 27.41% of missing data. Because our study analyzed secondary data, ethical approval was not necessary.

### Measures

Participants were assessed at school by trained researchers. Measures described below are part of a larger assessment battery (Norbury et al., 2016).

#### Irregular Word Reading

Irregular word reading was measured using the irregular word subtest from the Castles and Coltheart reading test 2 (CC2; Castles et al., 2009). Forty regular words, 40 irregular words, and 40 nonwords were presented on cards to participants in a mixed fashion. The words were presented in a fixed order so that easier words were presented earlier than more difficult words. Participants were instructed to read aloud the words. Once five consecutive errors were made for a word type (regular, irregular, or nonwords), the subtest for that word type was discontinued. Accuracy score for the irregular word subtest was used as an index of irregular word reading ability. Cronbach’s α was reported to be .86 (Moore et al., 2012).

#### Decoding

Decoding was measured by accuracy score on the CC2 nonwords subtest, which was administered using the same procedure as for the irregular word subtest. Cronbach’s α was reported to be .94 (Moore et al., 2012).

#### Vocabulary

Vocabulary was measured by the Receptive One-Word Picture Vocabulary Test (ROWPVT; Martin & Brownell, 2010) and the Expressive One-Word Picture Vocabulary Test (EOWPVT; Martin & Brownell, 2011). In ROWPVT, participants heard a word and were instructed to select the corresponding picture from four choices. In EOWPVT, they were instructed to name objects, actions, or concepts illustrated in pictures. According to the manual, Cronbach’s α and test–retest reliability coefficients for both measures are above .90.

#### Inhibitory Control

Inhibitory control was measured by a Go/No-Go task (Gooch et al., 2016). Participants were instructed to press a response key as quickly as possible when the Go stimulus (bug) appeared but to inhibit their response when the No-Go stimulus (ladybird) appeared. Each stimulus was preceded by a fixation cross and a varied lag (300, 600, or 900 ms). Participants completed eight practice trials followed by 80 randomized test trials (20 No-Go and 60 Go trials). Prior to the Go/No-Go test phase, participants completed 33 Go trials to establish the prepotent response, which were not included in the current analysis.

We planned to use six measures from the task to generate a latent variable of inhibitory control. Following Brocki and Bohlin (2004), these included (a) commission errors (responding to No-Go stimuli); (b) impulsivity errors (key pressing before the onset of stimulus); (c) mean reaction time (RT) in correct Go trials; and (d) omission errors (missing Go targets). Two additional measures were also used since they were proposed to be supported by the inhibitory control brain network (Bellgrove et al., 2004; Garavan et al., 2002): (e) the intraindividual variability (IIV) in RT (Dykiert et al., 2012), which was calculated as the ratio of the standard deviation of the individual’s RT to the mean of the individual’s RT in Go trials, and (f) the post error slowing (PES; Dutilh et al., 2012), calculated by averaging the RT difference between post error trials and pre error trials.

Using 5,000 random splits, the Spearman–Brown corrected reliability estimates (Parsons, 2021; Pronk et al., 2021) in Years 1, 3, and 6, respectively, were .71, .66, .72 for commission errors, .93, .89, .93 for impulsivity errors, .83, .74, .81 for omission errors, .86, .88, .90 for mean RT in correct trials, and .75, .77, .73 for IIV. The measurement of PES was based on consecutive trials with a certain pattern (correct Go trial, incorrect No-Go trial, correct Go trial). Therefore, trials were not split to estimate reliability for PES.

### Transparency and Openness

This study’s analysis plan and hypotheses were preregistered (https://osf.io/vah82). All data are available at the UK Data Service and can be accessed at https://doi.org/10.5255/UKDA-SN-8968-1. Analysis code has been made available on the Open Science Framework (https://osf.io/qkvyf/). Regarding the research materials, CC2 tests are openly available (Castles et al., 2009; https://www.motif.org.au/tests). ROWPVT and EOWPVT are only available from the publisher (Martin & Brownell, 2010, 2011). For access to the Go/No-Go task, please contact Gooch et al. (2016).

## Results

**Figure 3 fig3:**
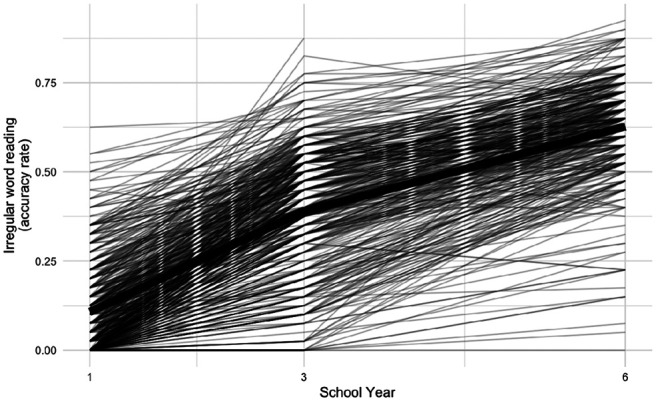
Developmental Trajectories of Irregular Word Reading Over Years 1, 3, and 6 *Note*. The trajectory in bold is based on means at each time point.

Descriptive statistics for observed measures in Years 1, 3, and 6 are shown in Table 1. Means increased over time for irregular word reading, decoding, and vocabulary measures, and decreased for inhibitory control measures (lower scores indicate better inhibitory control ability; cf., a lack of consensus for PES; Gupta et al., 2009; Wiersema et al., 2007). Developmental trajectories of irregular word reading are presented in Figure 3. Trajectories of other measures are available in Figures S1–S9 in the online supplemental materials.[Table tbl1][Fig fig3]

Structural equation modeling was conducted with Lavaan (Version 0.6-9; Rosseel, 2012) in R (Version 4.1.1). Robust full information maximum-likelihood estimation was used to account for missing data and deviations from normality (Shapiro–Wilk tests, *p*s < .01, in all measures across time points). Participants with complete data have better vocabulary knowledge than those with missing data. The two groups do not differ in terms of irregular word reading, decoding, or Go/No-Go measures (Table S1 in the online supplemental materials). The SCALES oversampled children with lower language abilities, which might have counteracted missing data from children with poor vocabulary knowledge.

### Latent Variables

A latent variable of vocabulary was constructed with two indicators, accuracy scores of both ROWPVT and EOWPVT. To construct the latent variable of inhibitory control, the covariance matrix between Go/No-Go measures was explored for each time point (Table S2 in the online supplemental materials). Commission error rate, IIV, and impulsivity error rate were intercorrelated across time points with coefficients between .43 and .62, indicating that their shared variance was explained by a common factor. Scatter plots (Figures S10–S18 in the online supplemental materials) showed that these intercorrelations were true effects and were not driven by outliers. No other cluster of measures was intercorrelated with effect sizes greater than .30 at any time point. Therefore, the commission error rate, IIV, and impulsivity error rate were used to construct the latent variable of inhibitory control. The standardized factor loadings are reported in Table 2.[Table tbl2]

Factorial invariance tests (Widaman et al., 2010) showed that the two latent variables changed over time in structure, as the model fit was significantly better when each parameter (indicator, factor loading, intercept) of the latent variables was freely estimated than when constrained to be equal across time points (*p*s < .05; lack of strong factorial invariance). This suggests that neither of the latent variables is strictly comparable across time points. Therefore, relations (correlations and regressions) that involve the latent variables are also not strictly comparable across time points and caution should be taken when interpreting any such comparisons. However, it should be noted that the main aim of the current study was to test whether there are effects of inhibitory control on irregular word reading and its growth at each time point, not to compare effect sizes over time. Therefore, the lack of strong factorial invariance did not affect our focal analysis.

### Predicting Irregular Word Reading

Before conducting regression analyses, correlations among all variables of interest were examined. All correlations were significant (*p*s < .01; Table 3). Irregular word reading significantly correlated with inhibitory control, as well as decoding and vocabulary, at each time point. Therefore, we proceeded to test whether inhibitory control predicts concurrent irregular word reading, after controlling for decoding and vocabulary.[Table tbl3]

#### Decoding and Vocabulary: Significant Predictors of Irregular Word Reading

For each time point, irregular word reading was first regressed on decoding and vocabulary. Robust fit indices (used throughout this study when reporting overall model fit) indicated that models had good fit at all time points (Schermelleh-Engel et al., 2003): Year 1, χ^2^(12) = 15.678, *p* = .206, comparative fit index (CFI) = .998, standardized root-mean-square residual (SRMR) = .014, root-mean-square error of approximation (RMSEA) = .024, 90% confidence interval (CI) = [0.000, 0.053]; Year 3, χ^2^(12) = 35.201, *p* < .001, CFI = .985, SRMR = .027, RMSEA = .065, [0.041, 0.090]; Year 6, χ^2^(12) = 15.545, *p* = .213, CFI = .997, SRMR = .024, RMSEA = .030, [0.000, 0.067].

The regressive paths from decoding and vocabulary were both significant across time points (*p*s < .001), with decreasing (.66, .58, .44) and increasing (.20, .34, .51) effect sizes, respectively. The increase in effect size in vocabulary should be taken with a degree of caution, as the vocabulary latent variable changed over time in structure (described earlier in the “Latent Variables” section). These analyses demonstrate that decoding and vocabulary significantly predicted concurrent irregular word reading in Years 1, 3, and 6, over and above each other.

#### Inhibitory Control: Not a Predictor of Irregular Word Reading Over and Above Decoding and Vocabulary

A regressive path from inhibitory control was then added to the model at each time point. Adding the path did not significantly improve fit at any time point: Year 1, Δχ^2^(1) = .173, *p* = .677; Year 3, Δχ^2^(1) = .122, *p* = .727; Year 6, Δχ^2^(1) = 1.120, *p* = .290, and the path was not significant at any time point, Year 1, *r* = −.013, *p* = .676; Year 3, *r* = .011, *p* = .73; Year 6, *r* = .042, *p* = .251. Therefore, after controlling for decoding and vocabulary, inhibitory control did not predict concurrent irregular word reading.

### Predicting Growth in Irregular Word Reading

**Figure 4 fig4:**
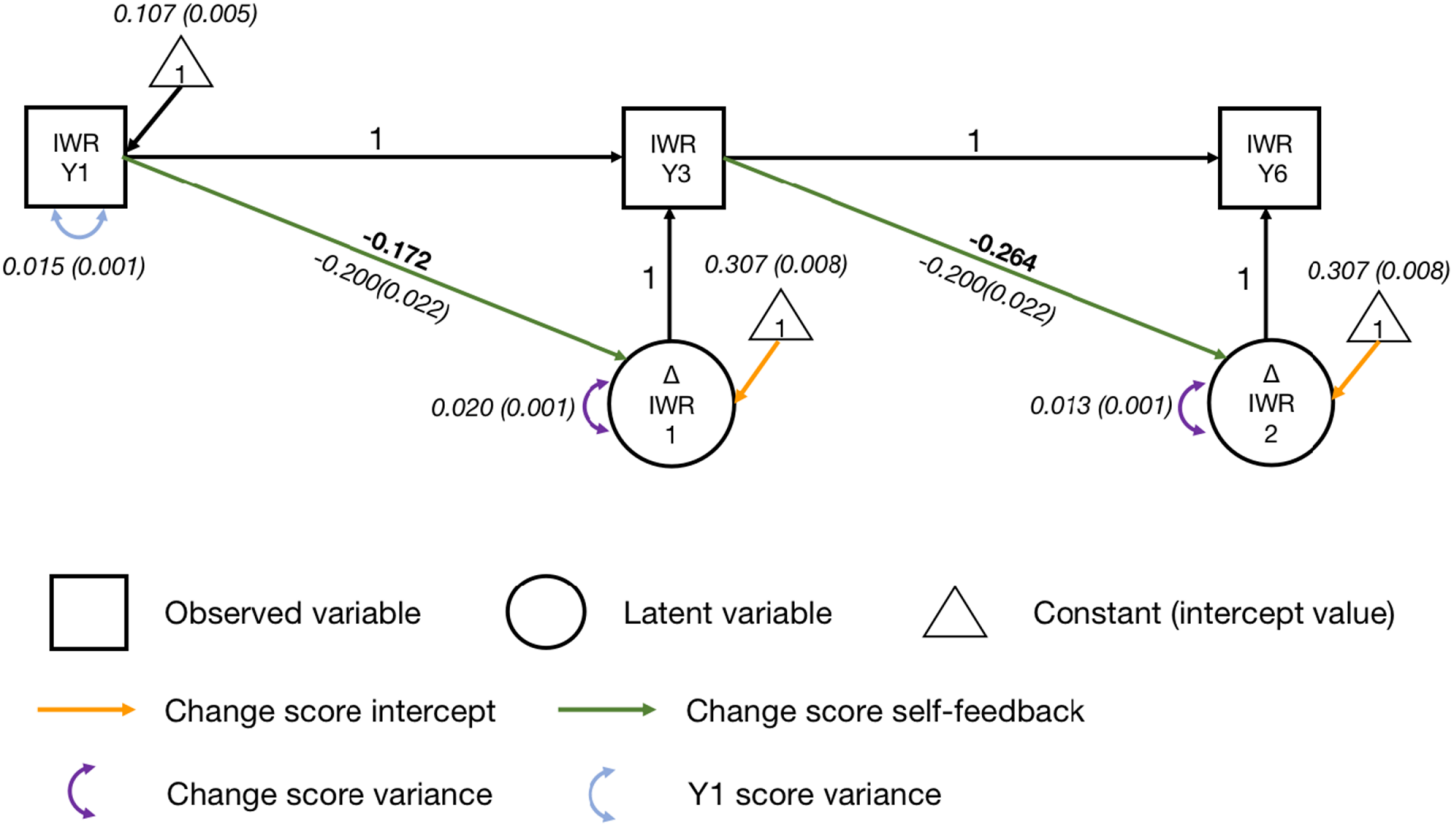
Univariate Latent Change Score Model of Irregular Word Reading *Note*. IWR = irregular word reading; Y1 = Year 1; Y3 = Year 3; Y6 = Year 6. Standardized parameter estimates are in roman font. Unstandardized parameter estimates (with standard error estimates in parentheses) are in italics. Key parameters of interest are in boldface. See the online article for the color version of this figure.

Before assessing what drives growth in irregular word reading, a univariate latent change score model (Figure 4; Kievit et al., 2018) was built for irregular word reading, with two latent change scores (circles with ΔIWR inside), one reflecting changes between Years 1 and 3, the other between Years 3 and 6. For the two change scores, each parameter was first constrained to be equal across waves (intercept, yellow arrows; variance, purple arrows; self-feedback parameter, green arrows). Variance parameters were then unconstrained, because this significantly improved fit, Δχ^2^(1) = 12.302, *p* < .001, as there was more variance in the change score between Years 1 and 3 than between Years 3 and 6.[Fig fig4]

The final model had a good fit, χ^2^(2) = 3.018, *p* = .221, CFI = .998, SRMR = .024, RMSEA = .029, 90% CI = [0.000, 0.090]. Initial irregular word reading ability significantly predicted change scores for irregular word reading in subsequent years (self-feedback effect; *p*s < .001; *r* = −.172, Year 1 reading predicting the change score between Years 1 and 3, *r* = −.264, Year 3 reading predicting the change score between Years 3 and 6). Participants with better initial irregular word reading ability achieved less growth in irregular word reading in the following years. After accounting for the effect of initial reading ability, a significant amount of variance remained to be explained in both change scores (*p*s < .001).

#### Inhibitory Control: A Significant Predictor of Growth

**Figure 5 fig5:**
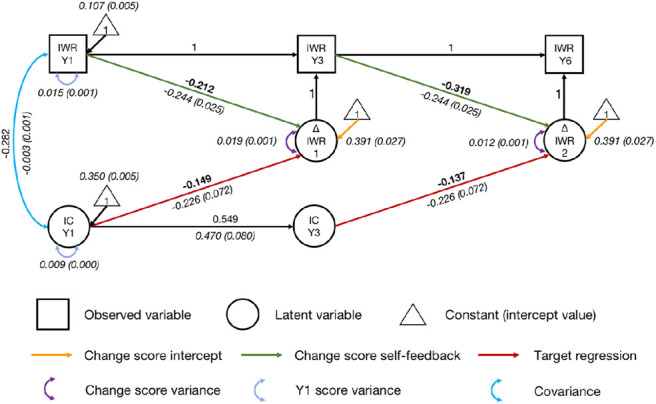
Univariate Latent Change Score Model of Irregular Word Reading Predicted by Inhibitory Control *Note*. IWR = irregular word reading; IC = inhibitory control; Y1 = Year 1; Y3 = Year 3; Y6 = Year 6. Standardized parameter estimates are in roman font. Unstandardized parameter estimates (with standard error estimates in parentheses) are in italics. Key parameters of interest are in boldface. Indicators and parameters of inhibitory control variables are not displayed for visual simplicity. See the online article for the color version of this figure.

Inhibitory control variables and two regressive paths were added to the univariate latent change score model, one path from Year 1 inhibitory control to the change score for irregular word reading between Years 1 and 3, and the other from Year 3 inhibitory control to the change score between Years 3 and 6 (Figure 5). The regressive parameters were constrained to be equal across waves where possible but were unconstrained if free estimation significantly improved model fit. Covariance was allowed between irregular word reading and inhibitory control in Year 1.[Fig fig5]

The final model had a good fit, χ^2^(23) = 52.522, *p* < .001, CFI = .983, SRMR = .050, RMSEA = .049, 90% CI = [0.032, 0.067]. After controlling for initial irregular word reading, both regressive paths from inhibitory control were significant (*p*s = .002). The effects were small (*r* = −.149, from Year 1 inhibitory control to the change score between Years 1 and 3; *r* = −.137, from Year 3 inhibitory control to the change score between Years 3 and 6). Participants with better inhibitory control ability gained more growth in irregular word reading. Fixing the regressive paths to zero significantly worsened model fit, Δχ^2^(1) = 8.881, *p* = .003. This confirms that inhibitory control contributed to growth in irregular word reading across time points.

#### Vocabulary: A Significant Predictor of Growth

**Figure 6 fig6:**
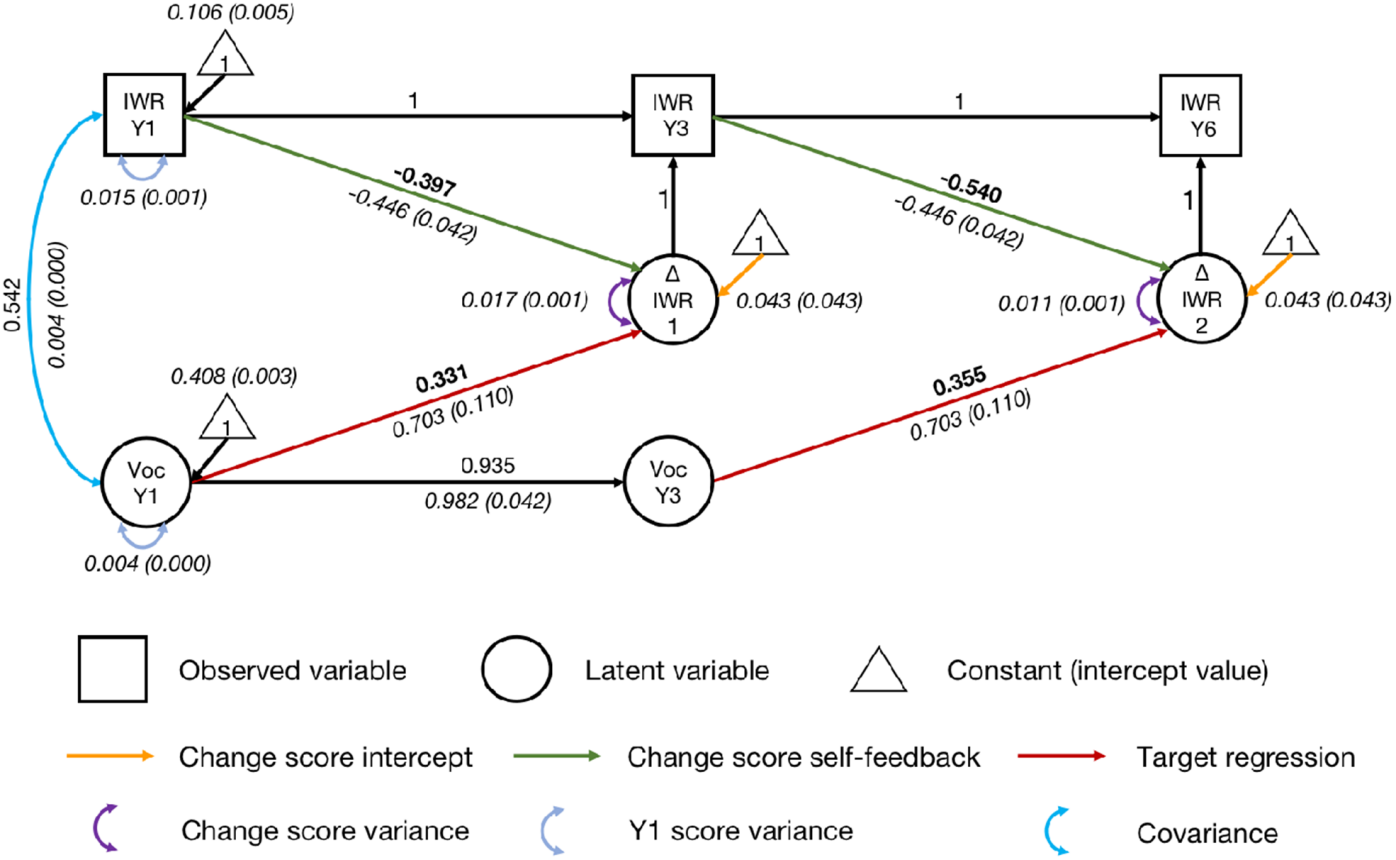
Univariate Latent Change Score Model of Irregular Word Reading Predicted by Vocabulary *Note*. IWR = irregular word reading; Voc = vocabulary; Y1 = Year 1; Y3 = Year 3; Y6 = Year 6. Standardized parameter estimates are in roman font. Unstandardized parameter estimates (with standard error estimates in parentheses) are in italics. Key parameters of interest are in boldface. Indicators and parameters of vocabulary variable are not displayed for visual simplicity. See the online article for the color version of this figure.

Similar analysis steps were applied for vocabulary, with two regressive paths from vocabulary (Figure 6). The final model had an acceptable fit, χ^2^(13) = 89.706, *p* < .001, CFI = .966, SRMR = .063, RMSEA = .103, 90% CI = [0.084, 0.124]. After controlling for initial irregular word reading ability, both regressive paths from vocabulary were significant (*p*s < .001). Participants with better vocabulary knowledge achieved more growth in irregular word reading, and the effects were medium (*r* = .331, from Year 1 vocabulary to the change score between Years 1 and 3; *r* = .355, from Year 3 vocabulary to the change score between Years 3 and 6). Fixing the regressive paths to zero significantly reduced fit, Δχ^2^(1) = 49.395, *p* < .001. This confirms that vocabulary significantly contributed to growth in irregular word reading across time points.[Fig fig6]

#### Decoding: Not a Predictor of Growth

Similar analysis steps were applied for decoding, with two regressive paths from decoding. The fit was unacceptable, both for the model with equality constraints, χ^2^(6) = 313.514, *p* < .001, CFI = .780, SRMR = .126, RMSEA = .312, 90% CI = [0.283, 0.342], and for the model without, χ^2^(5) = 312.337, *p* < .001, CFI = .780, SRMR = .129, RMSEA = .342, [0.310, 0.375]. Removing the regressive paths from decoding to the change scores significantly improved the fit, Δχ^2^(1) = 164.64, *p* < .001, such that the fit became acceptable, χ^2^(5) = 72.99, *p* < .001, CFI = .959, SRMR = .063, RMSEA = .148, [0.119, 0.179]. This demonstrates that the regressive paths from decoding were superfluous, that is, decoding did not predict growth in irregular word reading at any time point.

#### Inhibitory Control and Vocabulary: Significant Predictors of Growth Over and Above Each Other

**Figure 7 fig7:**
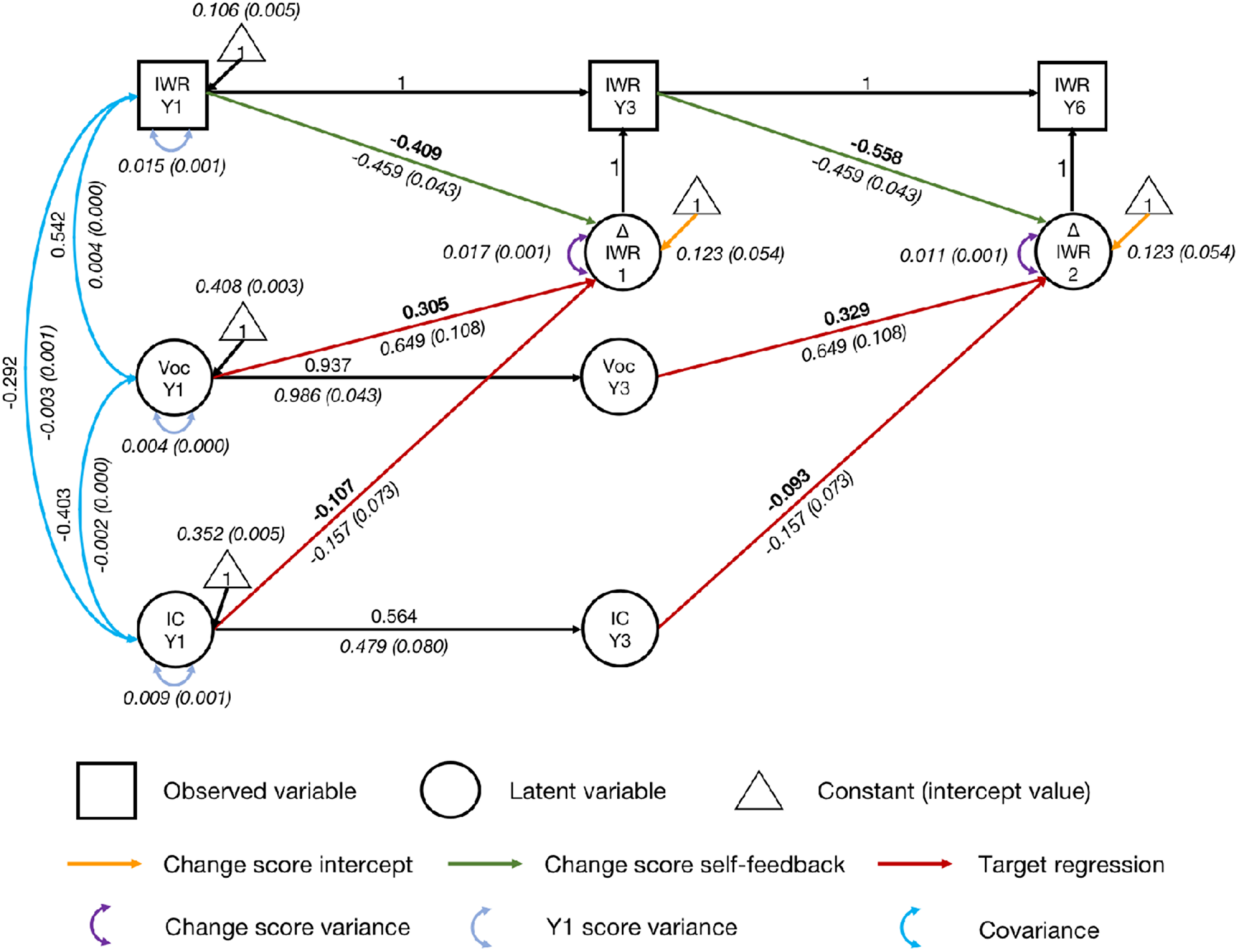
Univariate Latent Change Score Model of Irregular Word Reading Predicted by Vocabulary and Inhibitory Control *Note*. IWR = irregular word reading; Voc = vocabulary; IC = inhibitory control; Y1 = Year 1; Y3 = Year 3; Y6 = Year 6. Standardized parameter estimates are in roman font. Unstandardized parameter estimates (with standard error estimates in parentheses) are in italics. Key parameters of interest are in boldface. Indicators and parameters of vocabulary and inhibitory control latent variables are not displayed for visual simplicity. See the online article for the color version of this figure.

As shown in Figure 7, inhibitory control and vocabulary variables and their regressive paths were added to the univariate latent change score model (regressive paths from Year 1 inhibitory control and vocabulary to the latent change score for irregular word reading between Years 1 and 3, as well as from Year 3 inhibitory control and vocabulary to the latent change score for irregular word reading between Years 3 and 6). Covariance was allowed between the variables in Year 1. The final model had an acceptable fit, χ^2^(57) = 186.485, *p* < .001, CFI = .962, SRMR = .068, RMSEA = .066, 90% CI = [0.055, 0.076].[Fig fig7]

Both regressive paths from vocabulary were significant (*p*s < .001). The effects were medium (*r* = .305, from Year 1 vocabulary to the change score between Years 1 and 3; *r* = .329, from Year 3 vocabulary to the change score between Years 3 and 6). Fixing the regressive paths to zero for vocabulary significantly worsened fit, Δχ^2^(1) = 42.87, *p* < .001. These results suggest that vocabulary significantly contributed to growth in irregular word reading, after accounting for initial irregular word reading ability and inhibitory control.

After controlling for vocabulary and initial irregular word reading, regressive paths from inhibitory control were also significant (*p*s = .03). The effects were small (*r* = −.107, from Year 1 inhibitory control to the change score between Years 1 and 3; *r* = −.093, from Year 3 inhibitory control to the change score between Years 3 and 6). Fixing the regressive paths to zero for inhibitory control significantly reduced fit, Δχ^2^(1) = 4.406, *p* = .036. This provides evidence that inhibitory control contributed to growth in irregular word reading over and above vocabulary and initial irregular word reading, although the effects were small.

### Exploratory Analyses: Predicting Growth in Regular Word Reading

**Figure 8 fig8:**
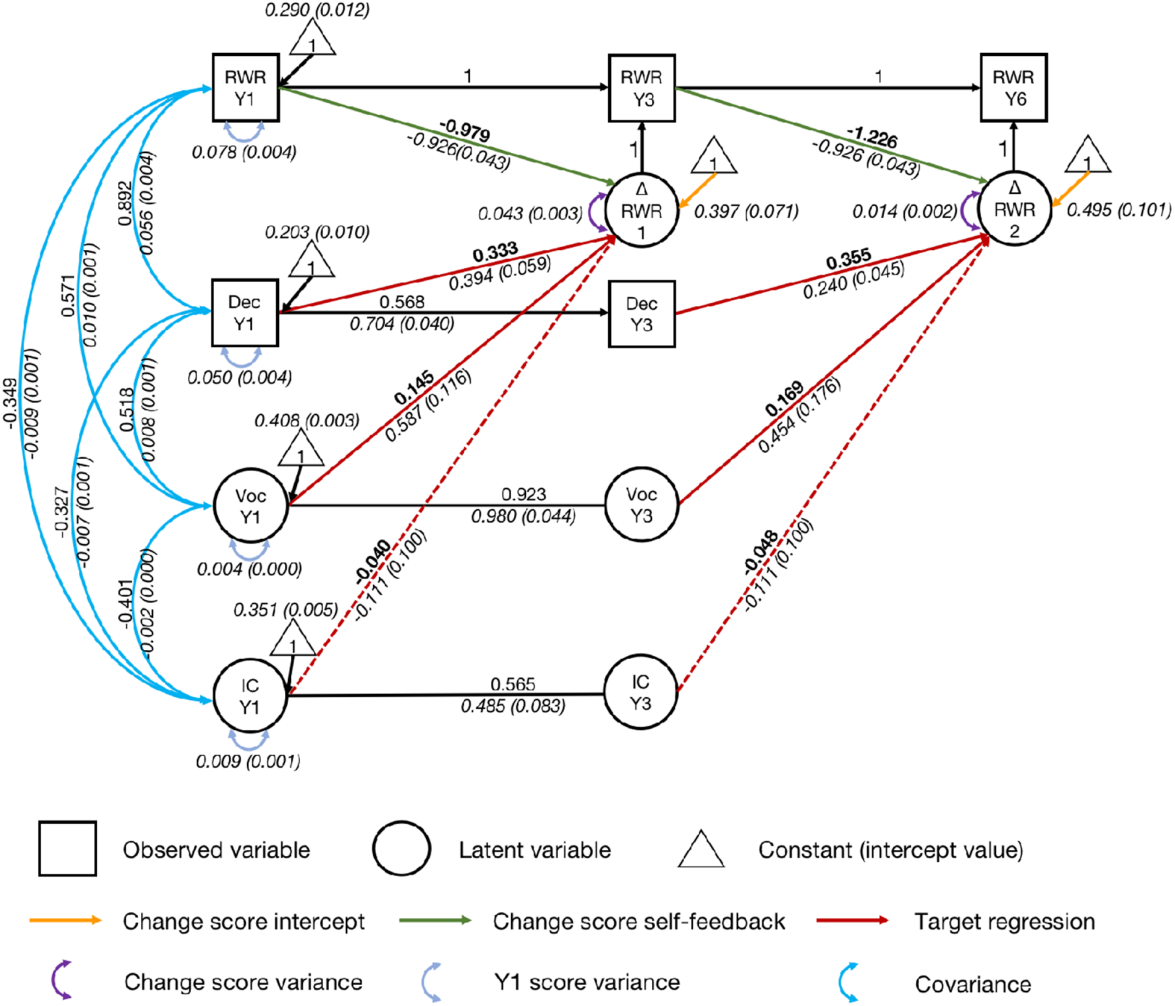
Univariate Latent Change Score Model of Regular Word Reading Predicted by Decoding and Vocabulary *Note*. RWR = regular word reading; Dec = decoding; Voc = vocabulary; IC = inhibitory control; Y1 = Year 1; Y3 = Year 3; Y6 = Year 6. Standardized parameter estimates are in roman font. Unstandardized parameter estimates (with standard error estimates in parentheses) are in italics. Key parameters of interest are in boldface. For visual simplicity, indicators, and parameters of vocabulary and inhibitory control latent variables are not displayed. The residual covariance between Year 3 decoding and the change score between Year 1 and 3 is also not displayed. See the online article for the color version of this figure.

As suggested by a reviewer, we conducted analyses to explore whether the role of inhibitory control is specific to growth in irregular word reading or more general so that it also predicts growth in regular word reading. As shown in Figure 8, inhibitory control, decoding, vocabulary, and their regressive paths were added to the univariate latent change score model of regular word reading (regressive paths from Year 1 inhibitory control, decoding, and vocabulary to the latent change score for regular word reading between Years 1 and 3, as well as from Year 3 inhibitory control, decoding, and vocabulary to the latent change score between Years 3 and 6). Covariances were allowed between Year 1 variables. The residual covariance between Year 3 decoding and the change score between Years 1 and 3 was added to the model.[Fig fig8]

The model had an acceptable fit, χ^2^(75) = 286.930, *p* < .001, CFI = .957, SRMR = .096, RMSEA = .074, 90% CI = [0.065, 0.084]. Initial regular word reading ability significantly predicted growth in regular word reading in subsequent years, with large effect sizes (*p*s < .001; *r* = −.979, Year 1 reading predicting the change score between Years 1 and 3; *r* = −1.226, Year 3 reading predicting the change score between Years 3 and 6). Participants with better initial regular word reading ability achieved less growth in regular word reading in the following years.

Regressive paths from decoding were significant (*p*s < .001). The effects were medium (*r* = .333, from Year 1 decoding to the change score between Years 1 and 3; *r* = .355, from Year 3 decoding to the change score between Years 3 and 6). Participants with better decoding ability achieved more growth in regular word reading. Fixing the regressive paths to zero significantly worsened fit, Δχ^2^(2) = 57.804, *p* < .001. Therefore, decoding significantly contributed to growth in regular word reading, after accounting for other predictors in the model. Regressive paths from vocabulary were also significant (*p*s < .001). The effects were small (*r* = .145, from Year 1 vocabulary to the change score between Years 1 and 3; *r* = .169, from Year 3 vocabulary to the change score between Years 3 and 6). Fixing the regressive paths to zero significantly worsened fit, Δχ^2^(2) = 30.218, *p* < .001. These results suggest that vocabulary also significantly contributed to growth in regular word reading, after controlling for other predictors in the model. Participants with better vocabulary knowledge achieved more growth in regular word reading.

After controlling for initial regular word reading, decoding, and vocabulary, inhibitory control did not significantly predict growth in regular word reading (*p*s = .265). Fixing the regressive paths to zero for inhibitory control did not worsen model fit, Δχ^2^(1) = 1.179, *p* = .278. Therefore, inhibitory control did not contribute to growth in regular word reading over and above decoding, vocabulary, and initial regular word reading. Full analyses and results for regular word reading are in the online supplemental materials (including descriptive statistics, developmental trajectories, a correlation matrix, regression analyses for concurrent regular word reading, and latent change score models for growth in regular word reading).

## Discussion

This study investigated the role of inhibitory control in irregular word reading and its development. It also tested whether and to what extent decoding and vocabulary predict growth in irregular word reading. We found that, although inhibitory control significantly correlated with concurrent irregular word reading at all time points, it did not predict concurrent irregular word reading at any time point after accounting for decoding and vocabulary. However, inhibitory control did predict growth in irregular word reading, even after controlling for vocabulary and initial irregular word reading (decoding did not predict the growth).

### Irregular Word Reading

#### Inhibitory Control: Not a Predictor of Irregular Word Reading Over and Above Decoding and Vocabulary

Consistent with the first hypothesis and data from human readers (Treiman et al., 1995), inhibitory control significantly correlated with concurrent irregular word reading across time points. However, after controlling for decoding and vocabulary, inhibitory control did not predict concurrent irregular word reading at any time point. This finding is inconsistent with our second hypothesis as well as our prediction from the DRC model (Coltheart et al., 2001), that is, inhibitory control should make a unique contribution to irregular word reading over and above decoding and vocabulary.

There are two possible reasons for the inconsistency. The first is statistical. At all time points, irregular word reading was highly correlated with concurrent decoding (*r* = .76, .77, .72 in Years 1, 3, 6, respectively) and vocabulary (*r* = .55, .66, .74 in Years 1, 3, 6, respectively). This might have soaked up variation in irregular word reading and left little variation for inhibitory control to explain. Relatedly, and replicating previous studies (Ober et al., 2020; Weiland et al., 2014), inhibitory control was significantly correlated with both covariates across time points (with decoding, −.32, −.38, −.31; with vocabulary, −.39, −.43, −.41, in Years 1, 3, and 6, respectively). During decoding (reading nonwords), readers might inhibit less common but still competing letter–sound mappings (Ulicheva et al., 2021). Processing vocabulary might also involve inhibition of potentially interfering distractors (Dagenbach et al., 1990). This could in part explain why inhibitory control did not contribute to concurrent irregular word reading once decoding and vocabulary (and any potential contributions of inhibitory control to these components) are accounted for. Nonetheless, the DRC model (Coltheart et al., 2001) suggests that inhibitory control should still make an additional contribution to irregular word reading, over and above decoding and vocabulary (and potential contributions of inhibitory control to these components).

The second reason for failing to find the effect is theoretical. The role of inhibitory control in concurrent irregular word reading might be word-specific, depending on readers’ familiarity with the word. In the DRC model (Coltheart et al., 2001), very familiar written words (e.g., high-frequency words like “break”) are highly activated along the lexical route, thus rapidly reaching the reading aloud threshold and leaving little time for the sublexical route to interfere. Therefore, there is little need to inhibit the decoded phoneme output. Conversely, when reading unfamiliar words (e.g., “zealot”), for which the lexical route is not established, there will be no lexical output. Readers can only decode the word using GPCs along the sublexical route and read aloud the decoded output, regardless of inhibitory control ability.

Thus, only for irregular words with intermediate familiarity will the lexical and sublexical route be activated to a similar extent, such that readers need to inhibit the predisposition to read aloud the decoded output and settle on the lexical output instead. The current study collapsed across irregular words with various degree of familiarity and might have masked the effect of inhibitory control on reading irregular words with intermediate familiarity. Future research can investigate whether inhibitory control makes different contributions to reading of irregular words with varying degrees of familiarity.

#### Decoding and Vocabulary: Significant Predictors of Irregular Word Reading

Consistent with the DRC model (Coltheart et al., 2001) and previous empirical studies (Johnston et al., 2014; Ricketts et al., 2007; Steacy et al., 2017), decoding and vocabulary significantly predicted concurrent irregular word reading across time points, after controlling for each other. Over time, the effect of decoding on concurrent irregular word reading decreased, and that of vocabulary increased. This suggests that, with increasing age and reading experience, decoding is less important, whereas vocabulary becomes more important for reading irregular words. However, as described in the “Latent Variables” section, the vocabulary variable is not strictly comparable across time points, so this result should be taken with caution. Future studies could add one or more vocabulary tasks to better construct the vocabulary variable, and then compare effect sizes across time points.

### Predicting Growth in Irregular Word Reading

#### Inhibitory Control: A Significant Predictor of Growth Over and Above Vocabulary

Supporting the final hypothesis, inhibitory control contributed to the growth in irregular word reading across time points, over and above vocabulary and initial irregular word reading ability. The effects were small but significant. This provides empirical evidence for the ST-DRC model (Pritchard et al., 2018), which suggests that, in order to successfully self-teach irregular words, readers need to inhibit the predisposition to use the decoded output to establish the lexical route. Instead, readers should allow time to recognize the correct lexical output and use that to develop the correct lexical route. The finding is also consistent with evidence that children might need to inhibit the strong predisposition to use GPCs to encode a regularized orthographic representation when developing the lexical route (Wang et al., 2011, 2012). It should be noted that, though the current study interprets the role of inhibitory control in the development of irregular word reading under the self-teaching framework, children might learn irregular words through different approaches (e.g., self-teaching, sight word instruction, understanding the history of words; see Colenbrander et al., 2020). The extent to which inhibition demands vary between teaching approaches is one avenue for future studies.

To assess whether inhibitory control is important to general word reading development as suggested by a reviewer, we conducted exploratory analyses in which the outcome measure was growth in regular word reading. Results showed that decoding (medium effects) and vocabulary (small effects) made unique contributions to growth in regular word reading, but inhibitory control did not. While we cannot draw strong conclusions from the null effect of inhibitory control, our results are in line with the ST-DRC model (Pritchard et al., 2018), Murray et al. (2022) and Wang et al. (2011, 2012), which suggest that inhibitory control might be particularly important when readers need to inhibit the predisposition to overgeneralize the most common relations between letters and sounds during self-teaching of irregular words.

#### Vocabulary but Not Decoding Predicted the Growth

Previous theoretical (Share, 1995; the ST-DRC model, Pritchard et al., 2018) and empirical work (Nation & Snowling, 2004; Ricketts et al., 2007) suggests that decoding and vocabulary are important for the development of irregular word reading. The current study found that vocabulary predicted growth in irregular word reading at both time points with medium effects, after controlling for other factors. This provides empirical evidence that accessing vocabulary knowledge in the mental lexicon helps develop correct connections between print and sound for irregular words.

However, decoding did not predict growth in irregular word reading in our participants, which is inconsistent with previous research. Possible reasons are threefold. First, the current study used a much larger sample size (*N* = 529) than previous studies (*N* = 72, Nation & Snowling, 2004; *N* = 30, Ricketts et al., 2007), and therefore had more power to detect true effects and avoid spurious results. Second, previous research (Nation & Snowling, 2004; Ricketts et al., 2007) tested whether decoding predicts irregular word reading at a later time point. However, the current study used latent change score models, which extracted the irregular word reading growth variable and allowed more specific assessment of whether decoding predicts this growth.

Third, decoding might not play such an important role in the development of irregular word reading once a certain level of decoding performance is reached. Simulation 2 of the ST-DRC model (Pritchard et al., 2018) showed that being able to decode single-letter GPCs (e.g., decode one-letter grapheme “b” to [b]) was sufficient to self-teach irregular words, when the context was available and ambiguous. Having further decoding skills (e.g., decoding multi-letter grapheme “ph” into [f]) made little difference in self-teaching outcomes. It is likely that the majority of our participants were capable of decoding single-letter GPCs, and only varied in further decoding skills (e.g., decoding multi-letter GPCs). Such a lack of variation in single-letter-GPC decoding ability might explain why decoding did not predict growth in irregular word reading in the current study. Future work could use artificial language learning paradigms (e.g., Taylor et al., 2011) to manipulate training and ensure variation in single-letter-GPC decoding ability, and then test whether decoding predicts growth in irregular word reading.

#### Implications

Given that inhibitory control and vocabulary predicted growth in irregular word reading, teachers may want to encourage students to inhibit the predisposition to use the decoded phoneme output (e.g., /tʃef/) without checking whether it fully matches with the pronunciation of known spoken words (/ʃef/). Students should decode and actively search for known spoken words that make sense in context, check potential mismatches between the decoded output (/tʃef/) and the lexical output (/ʃef/), and choose the lexical output (/ʃef/) if mismatches arise.

The latter idea (using the lexical output to correct the decoded output) is similar to the set for variability account. A set for variability refers to the metacognitive awareness that letter–sound relations can be variable (Gibson, 1965), and the strategy that, if the decoded output does not sound like a known spoken word, readers should try different pronunciations until they recognize a similar sounding word that makes sense in context (Venezky, 1999). Dyson et al. (2017) found that, compared to the control group, the intervention group who received training in the set for variability achieved significantly greater improvement in reading trained irregular words and untrained irregular words of equivalent difficulty to the trained words (but not in reading more difficult untrained irregular words). Therefore, it might be useful to teach the set for variability to help children learn to read irregular words. However, while the set for variability strategy seems to imply that readers should avoid using the decoded output, the relation between the set for variability and inhibitory control is not clear. Future research can investigate this relation by, for example, testing whether additional explicit training in inhibiting the decoded output will significantly enhance the set for variability training.

Additionally, given findings from studies of orthographic learning via self-teaching (Wang et al., 2011, 2012), teachers may want to remind students that if they generate a spelling based on their spoken word knowledge (e.g., /ʃef/), this encoded representation (e.g., SHEF) might be inaccurate and should be checked against the written form in print (e.g., CHEF).

### Generalizability of Our Findings

Our findings are based on analysis of data from monolingual English–speaking children whose schooling involved explicit teaching of letter–sound mapping rules (phonics). The role of inhibitory control, vocabulary, and decoding in irregular word reading and its development might differ for children who receive different literacy instruction (Castles et al., 2018). The current study focused on irregular words in English and findings might not generalize to writing systems with different regularity characteristics (Dehaene, 2010). More research is needed to understand the role of inhibitory control, vocabulary, and decoding in reading and learning to read irregular words in other writing systems.

## Conclusion

This study investigated the role of inhibitory control in irregular word reading and its development. It also tested whether and to what extent decoding and vocabulary predict growth in irregular word reading. Secondary data analysis was conducted on three-wave subsets of a longitudinal U.K. population-based project. We found that inhibitory control did not predict concurrent irregular word reading over and above the covariates of decoding and vocabulary. However, inhibitory control made a small but significant contribution to the growth in irregular word reading across time points, after controlling for vocabulary and initial irregular word reading ability. Additionally, vocabulary, but not decoding, made a medium and significant contribution to the growth in irregular word reading, after accounting for inhibitory control and initial irregular word reading ability. This research advances our understanding of how children read irregular words and achieve development in irregular word reading. It also has practical implications for teaching irregular words.

## Supplementary Material

10.1037/dev0001563.supp

## Figures and Tables

**Table 1 tbl1:** Descriptive Statistics for Observed Measures in Years 1, 3, and 6

	Year 1			Year 3			Year 6		
Measure	*N*	Mean	*SD*	*N*	Mean	*SD*	*N*	Mean	*SD*
Irregular word reading^a^	517	10.71%	12.33%	495	38.86%	17.30%	377	62.73%	14.29%
Decoding^a^	517	20.59%	22.27%	495	53.04%	27.77%	377	75.07%	23.24%
Vocabulary									
ROWPVT^a^	528	40.77%	7.44%	499	51.40%	7.74%	384	64.16%	10.23%
EOWPVT^a^	524	39.46%	7.93%	499	48.04%	8.21%	372	61.99%	9.34%
Inhibitory control (Go/No-Go)									
Mean RT^b^	508	593.15	83.26	490	515.41	67.32	381	417.02	56.18
IIV	508	0.35	0.11	489	0.30	0.10	381	0.28	0.09
Omission^c^	508	10.35%	8.06%	489	6.15%	5.97%	381	3.06%	4.85%
Commission^c^	508	33.95%	18.85%	489	28.88%	16.67%	381	23.48%	16.73%
Impulsivity^c^	512	7.32%	10.15%	491	4.07%	6.45%	381	2.63%	6.29%
PES^b^	465	130.72	206.10	455	92.85	160.60	339	73.74	141.38
*Note*. ROWPVT = receptive one-word picture vocabulary test; EOWPVT = expressive one-word picture vocabulary test; RT = reaction time; IIV = intraindividual variability in reaction time; PES = posterror slowing.									
^a^ Accuracy rate. ^b^ In millisecond. ^c^ Error rate.									

**Table 2 tbl2:** Standardized Factor Loadings of Latent Variables in Years 1, 3, and 6

Latent variable	Year 1	Year 3	Year 6
Inhibitory control			
IIV	0.79	0.78	0.75
Commission	0.69	0.63	0.71
Impulsivity	0.87	0.85	0.73
Vocabulary			
ROWPVT	0.88	0.90	0.86
EOWPVT	0.83	0.85	0.94
*Note*. IIV = intraindividual variability in reaction time; ROWPVT = receptive one-word picture vocabulary test; EOWPVT = expressive one-word picture vocabulary test.			

**Table 3 tbl3:** Correlation Matrix for Variables of Interest in Years 1, 3, and 6

Variables	1	2	3	4	5	6	7	8	9	10	11	12
1. Irregular word reading in Year 1	—											
2. Inhibitory control in Year 1	−.29	—										
3. Decoding in Year 1	.76	−.32	—									
4. Vocabulary in Year 1	.55	−.39	.53	—								
5. Irregular word reading in Year 3	.59	−.33	.50	.56	—							
6. Inhibitory control in Year 3	−.27	.56	−.31	−.40	−.35	—						
7. Decoding in Year 3	.55	−.30	.57	.47	.77	−.38	—					
8. Vocabulary in Year 3	.50	−.39	.45	.92	.66	−.43	.57	—				
9. Irregular word reading in Year 6	.50	−.29	.44	.57	.67	−.35	.61	.60	—			
10. Inhibitory control in Year 6	−.28	.42	−.29	−.42	−.38	.45	−.41	−.50	−.29	—		
11. Decoding in Year 6	.40	−.29	.43	.37	.61	−.36	.71	.47	.72	−.31	—	
12. Vocabulary in Year 6	.49	−.39	.44	.76	.56	−.45	.48	.79	.74	−.41	.55	—
