# Supplementary material for: Inhibitory Control Predicts Growth in Irregular Word Reading: Evidence From a Large-Scale Longitudinal Study
Source: Dev Psychol. 2023 Aug 31;59(12):2367–78. doi: 10.1037/dev0001563 (PMC10680298; doi:10.1037/dev0001563)
Supplement: Supplementary file 1 [file DEV-2022-0374_Supplemental_Materials.docx]

**Supplementary materials**

**Figure S1**

*Developmental trajectories of decoding over Years 1, 3 and 6*


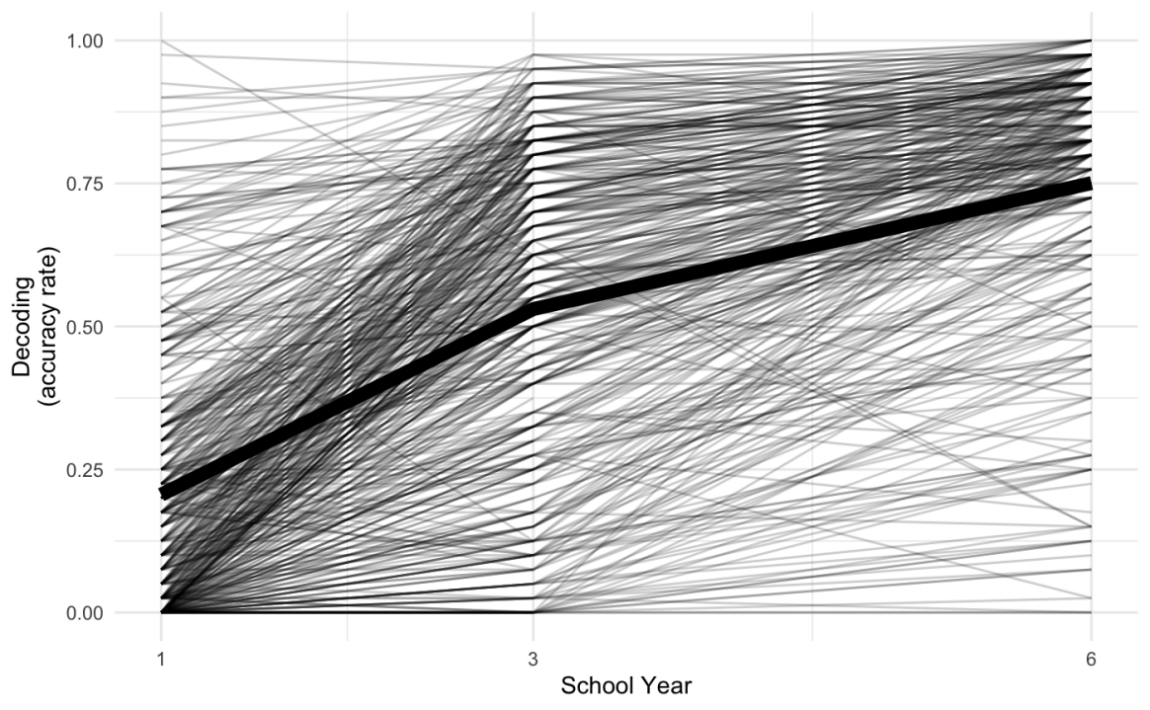


*Note*. The trajectory in bold is based on means at each time point.

**Figure S2**

*Developmental trajectories of receptive vocabulary over Years 1, 3 and 6*


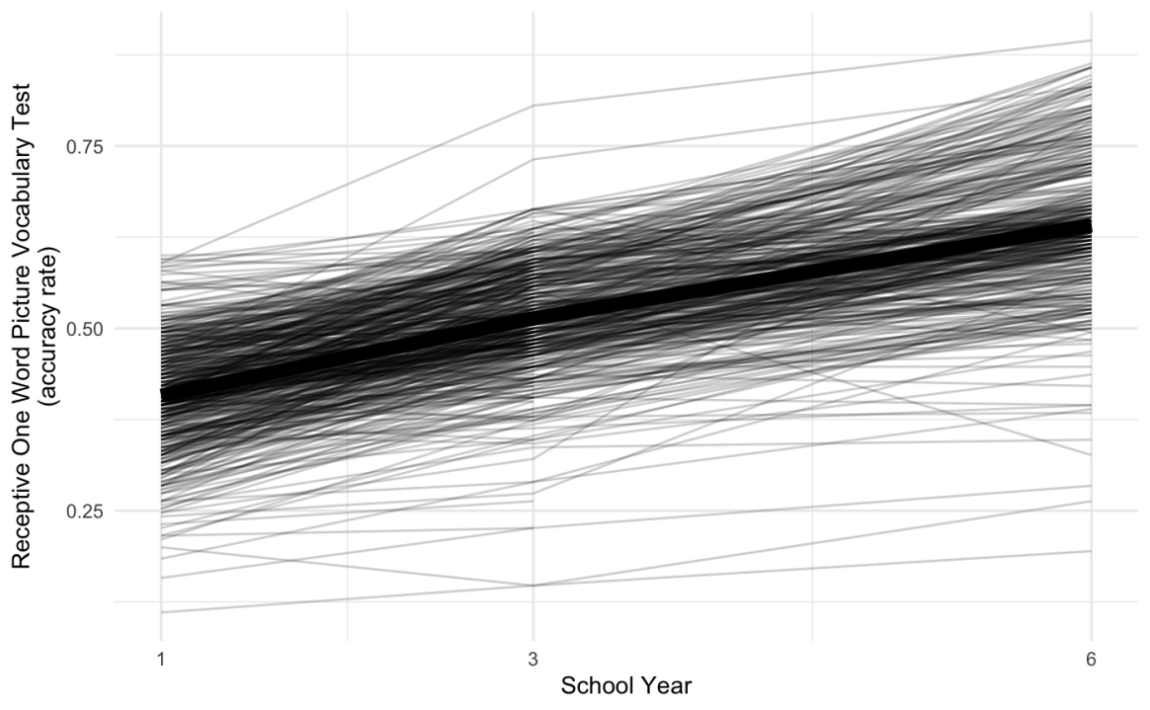


*Note*. The trajectory in bold is based on means at each time point.

**Figure S3**

*Developmental trajectories of expressive vocabulary over Years 1, 3 and 6*


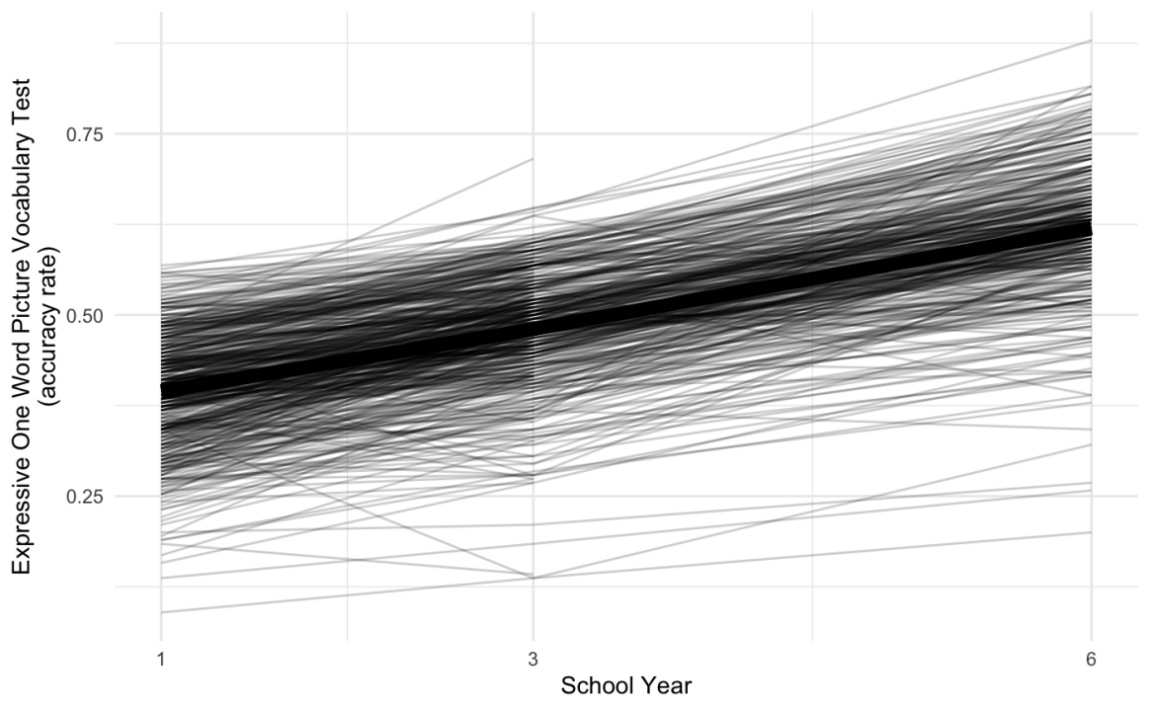


*Note*. The trajectory in bold is based on means at each time point.

**Figure S4**

*Developmental trajectories for mean reaction time in correct Go trials over Years 1, 3 and 6*


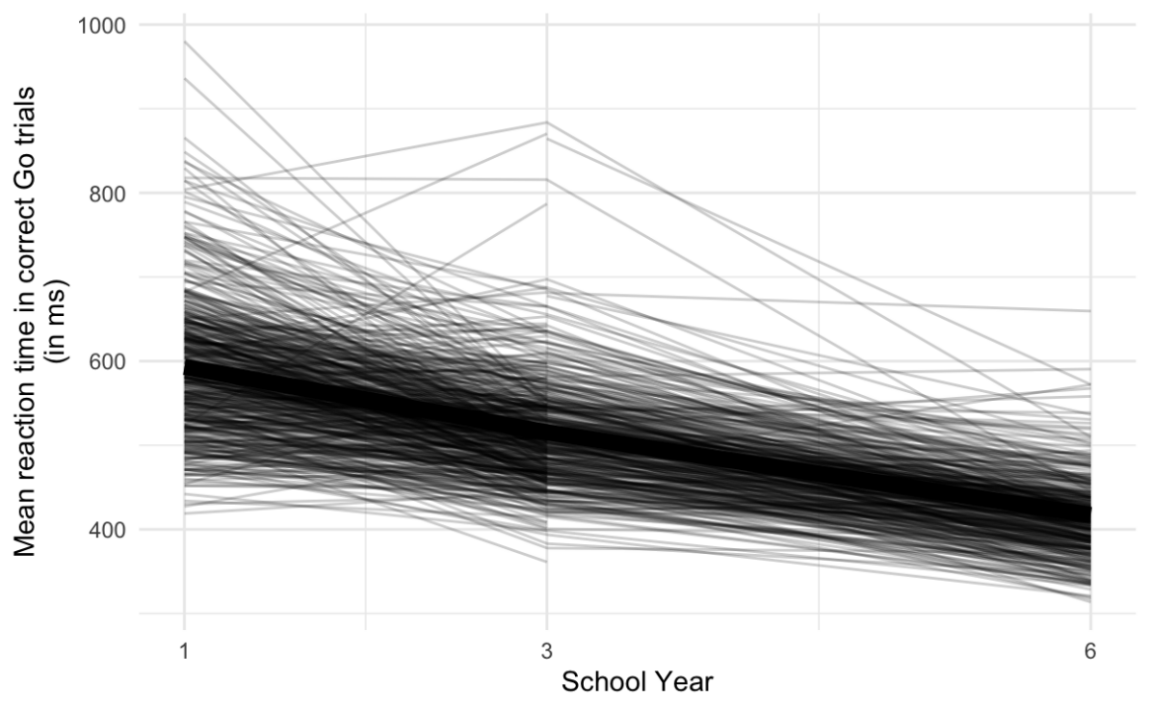


*Note*. The trajectory in bold is based on means at each time point.

**Figure S5**

*Developmental trajectories for intra-individual variability in reaction time over Years 1, 3 and 6*


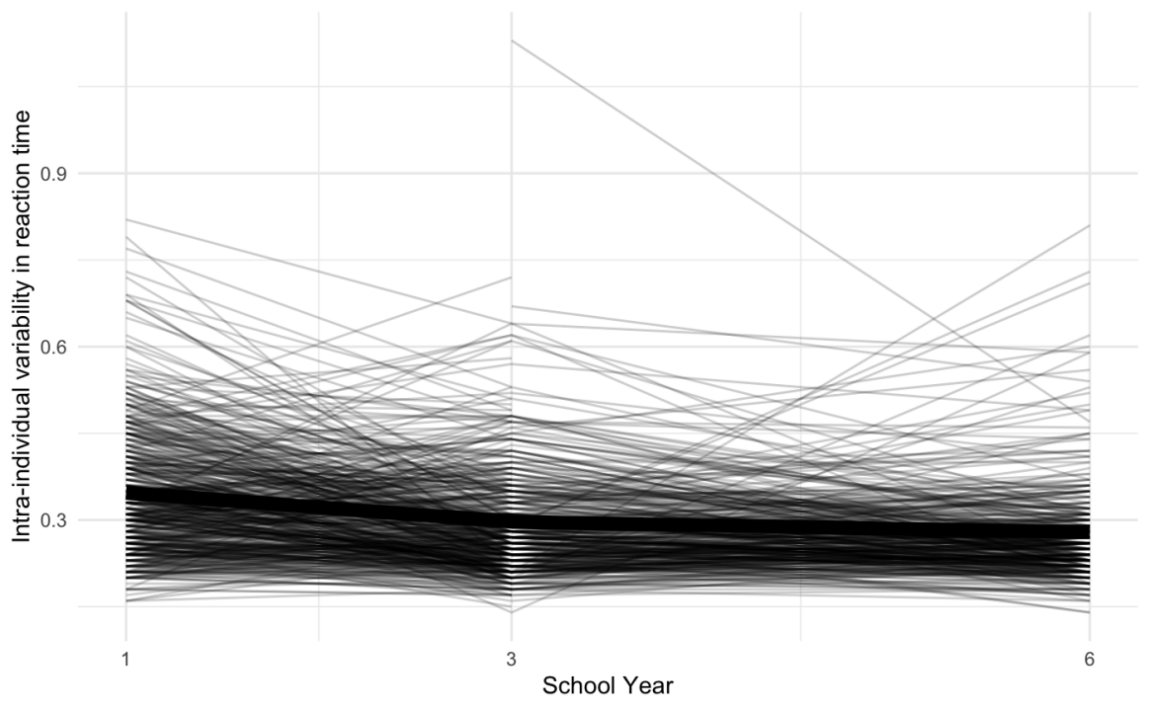


*Note*. The trajectory in bold is based on means at each time point.

**Figure S6**

*Developmental trajectories for omission error rate over Years 1, 3 and 6*


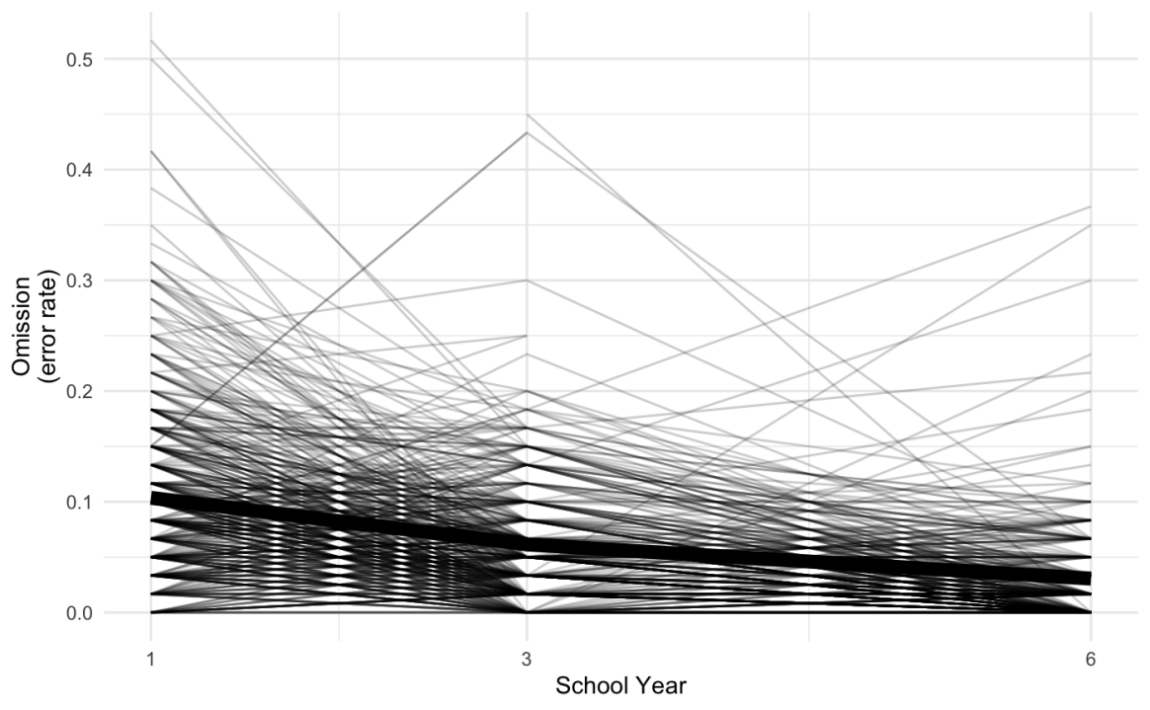


*Note*. The trajectory in bold is based on means at each time point.

**Figure S7**

*Developmental trajectories for commission error rate over Years 1, 3 and 6*


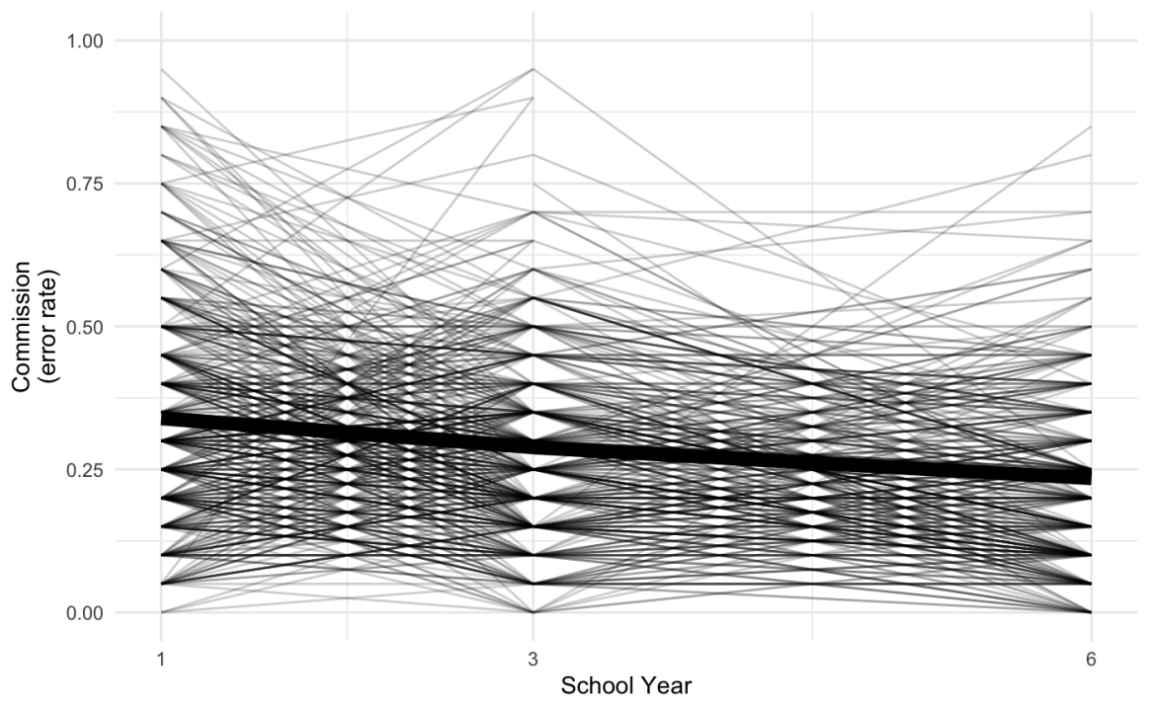


*Note*. The trajectory in bold is based on means at each time point.

**Figure S8**

*Developmental trajectories for impulsivity error rate over Years 1, 3 and 6*


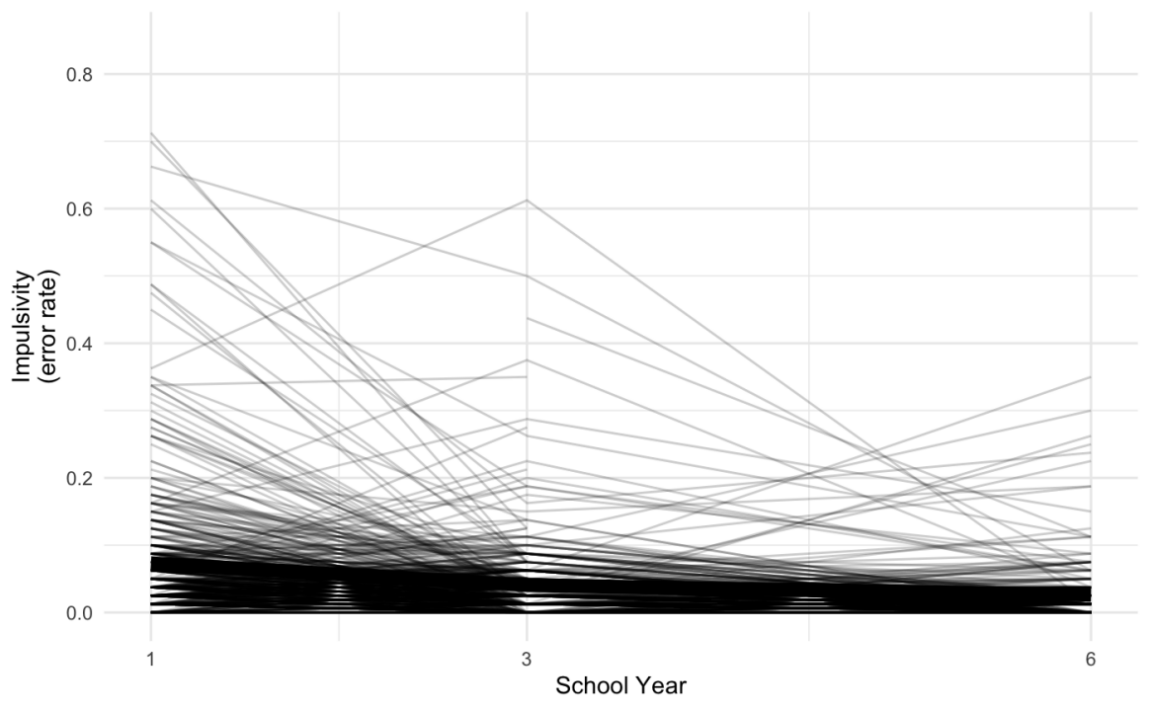


*Note*. The trajectory in bold is based on means at each time point.

**Figure S9**

*Developmental trajectories for post-error slowing over Years 1, 3 and 6*


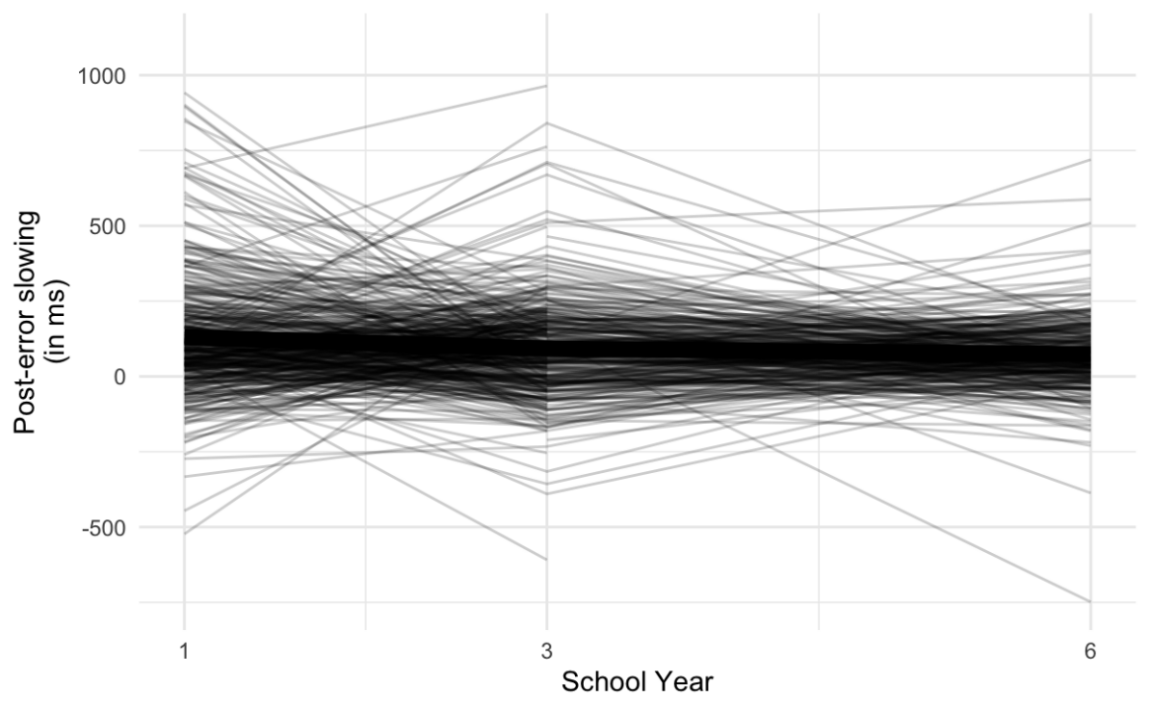


*Note*. The trajectory in bold is based on means at each time point.

**Figure S10**

*Scatter plot for impulsivity error rate and commission error rate in Year 1*

**
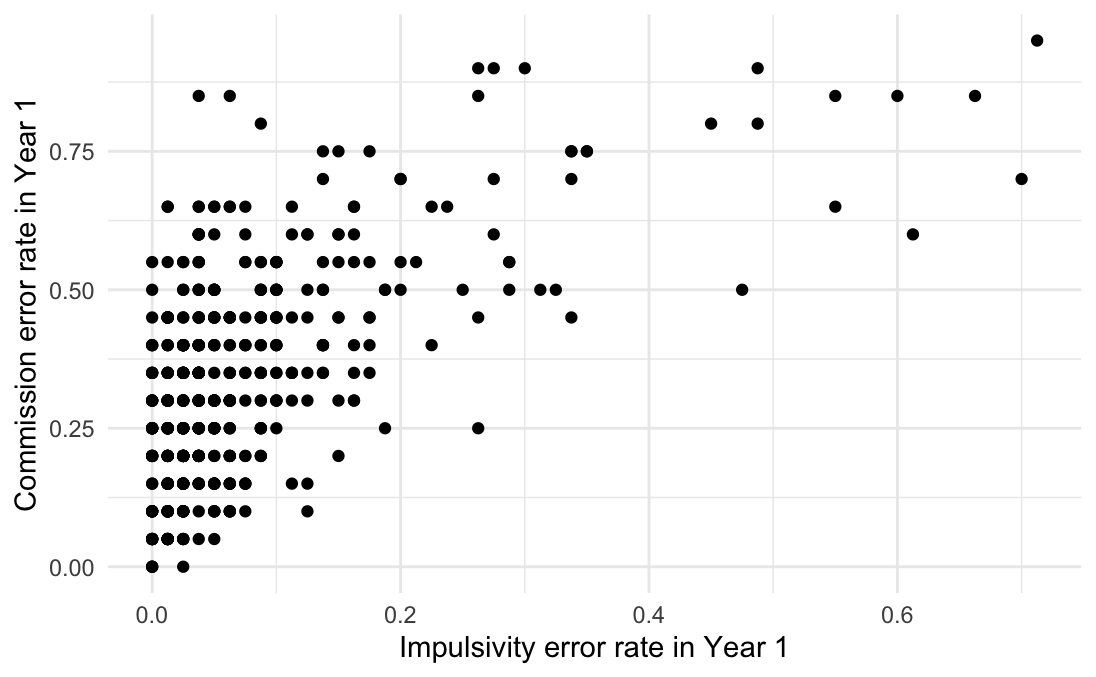
**

**Figure S11**

*Scatter plot for impulsivity error rate and intra-individual variability in reaction time in Year 1*

*
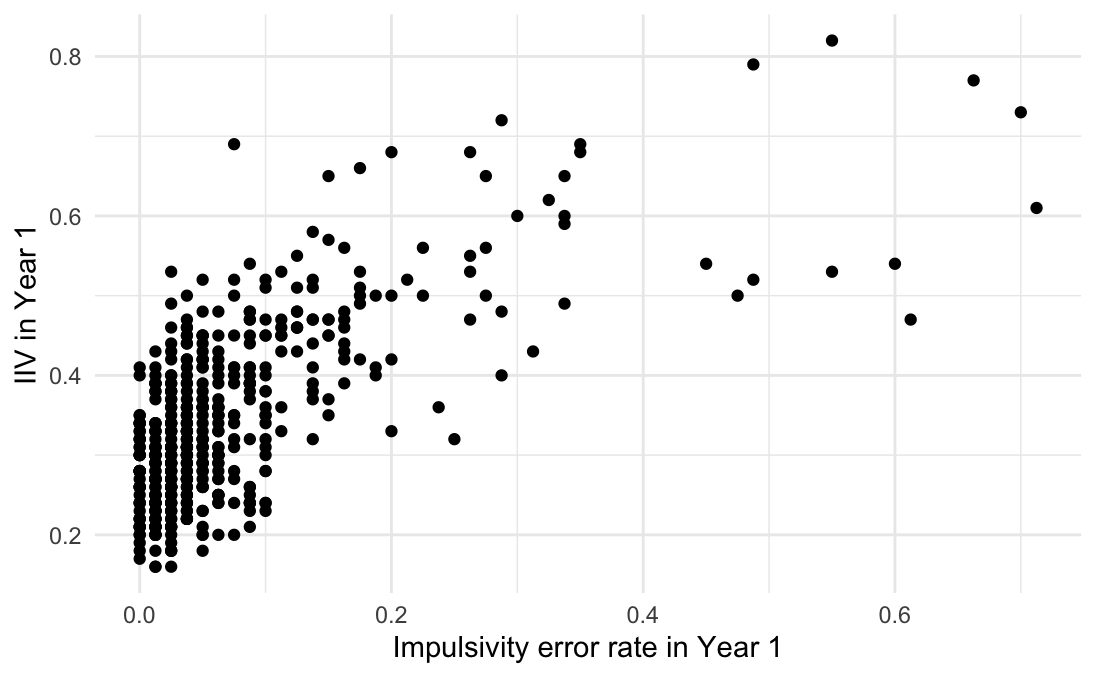
*

*Note*. IIV = intra-individual variability in reaction time.

**Figure S12**

*Scatter plot for commission error rate and intra-individual variability in reaction time in Year 1*

*
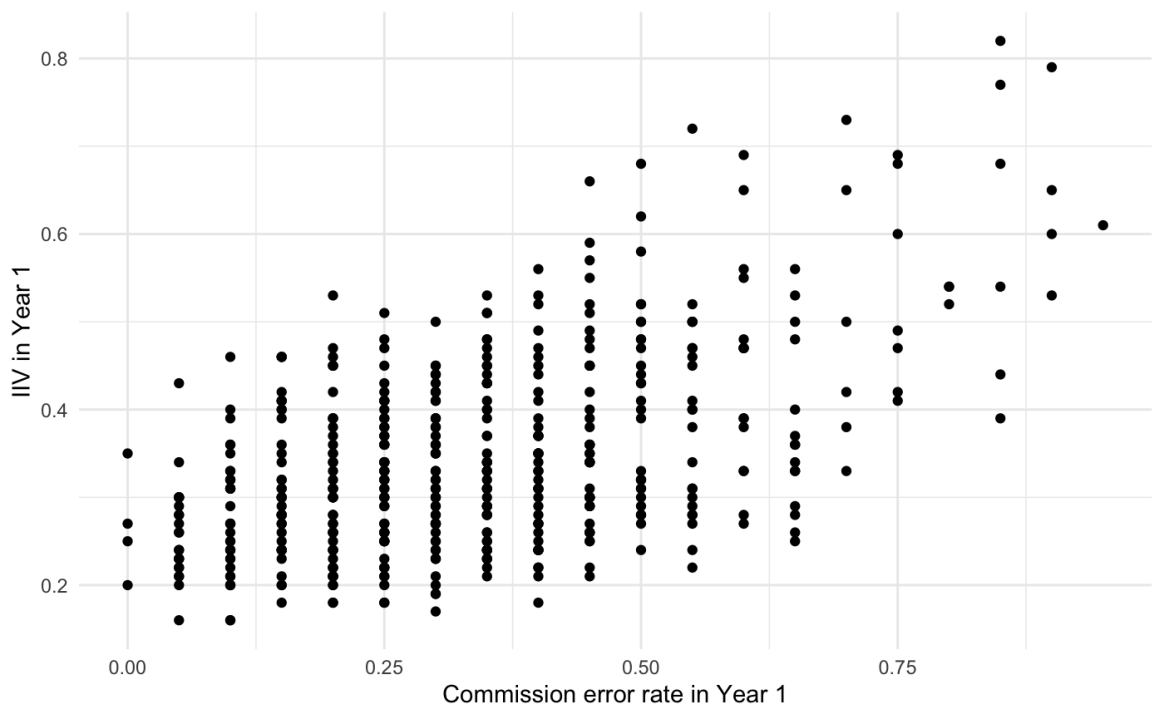
*

*Note*. IIV = intra-individual variability in reaction time.

**Figure S13**

*Scatter plot for impulsivity error rate and commission error rate in Year 3*


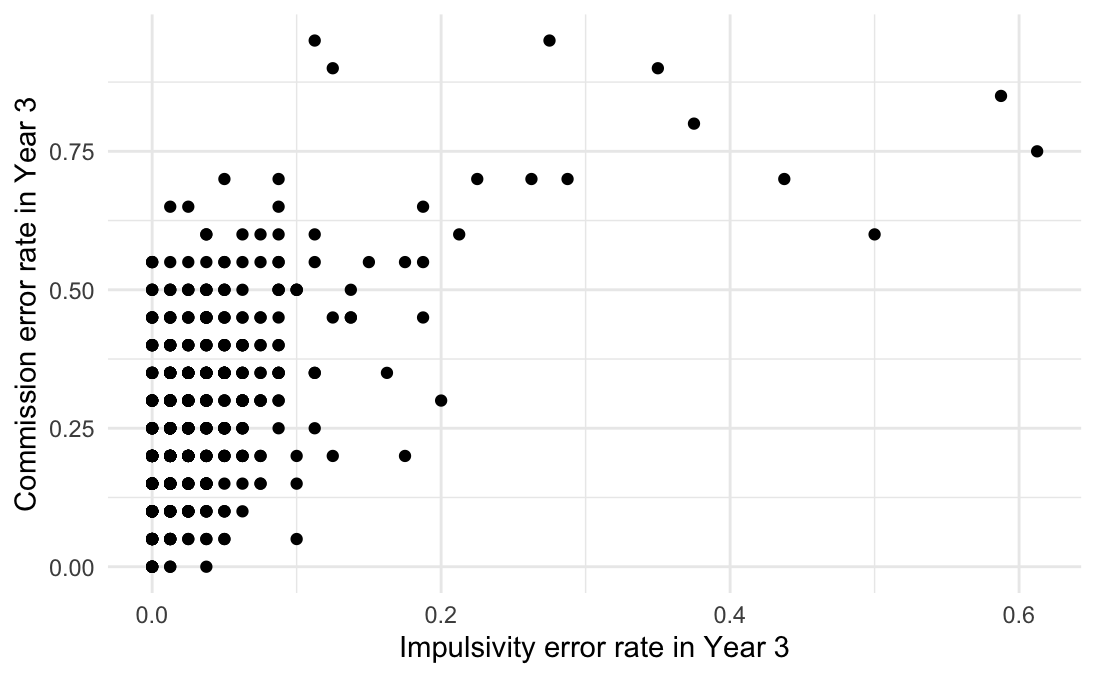


**Figure S14**

*Scatter plot for impulsivity error rate and intra-individual variability in reaction time in Year 3*


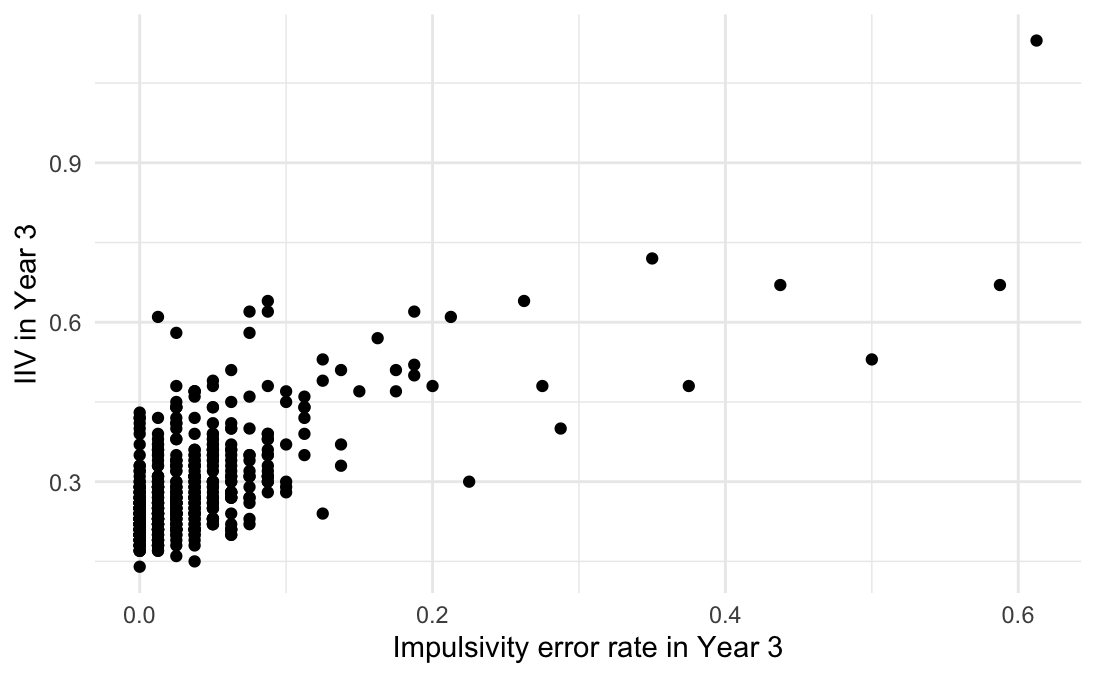


*Note*. IIV = intra-individual variability in reaction time.

**Figure S15**

*Scatter plot for commission error rate and intra-individual variability in reaction time in Year 3*

*
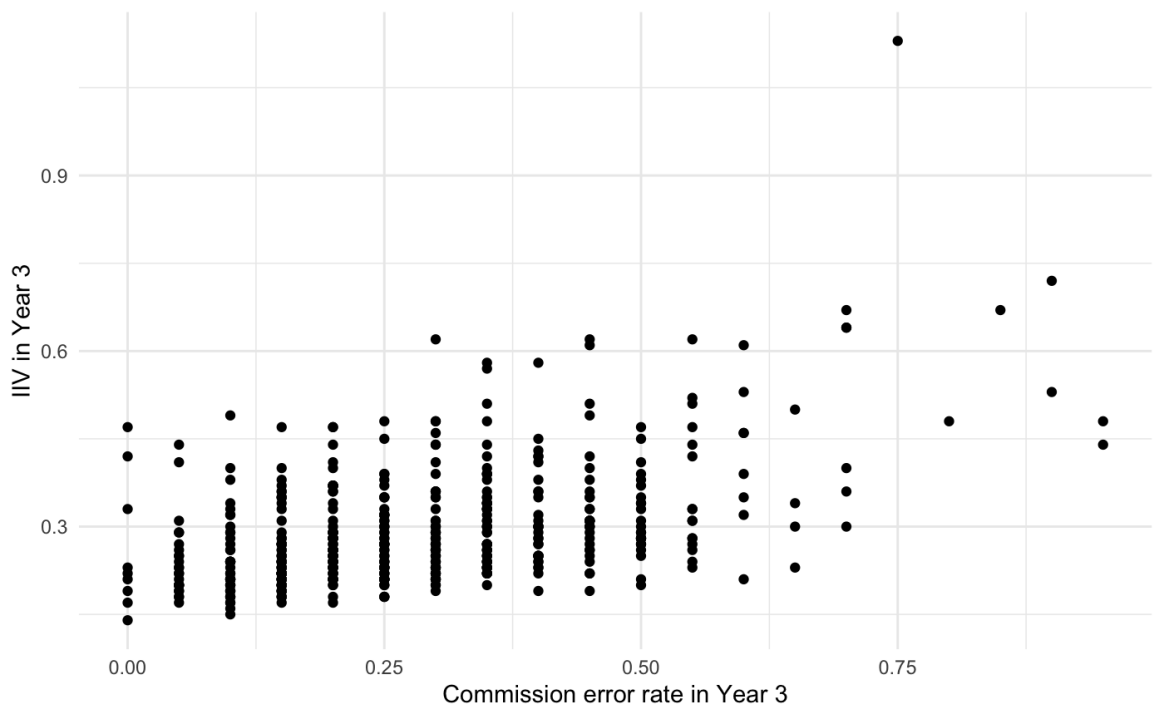
*

*Note*. IIV = intra-individual variability in reaction time.

**Figure S16**

*Scatter plot for impulsivity error rate and commission error rate in Year 6*


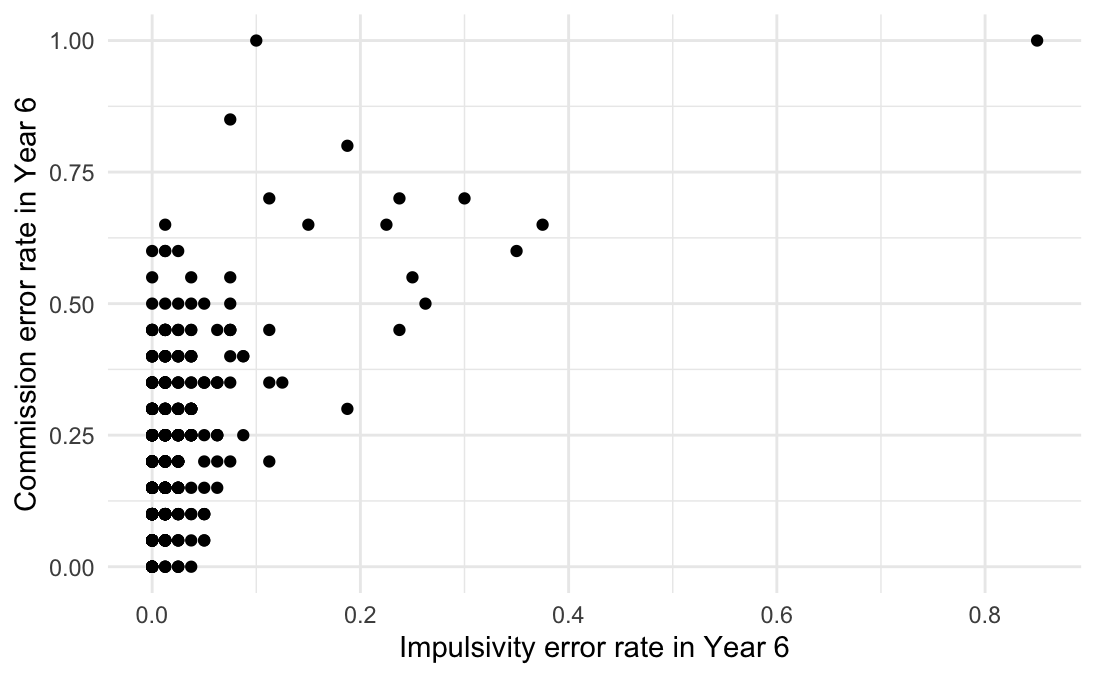


**Figure S17**

*Scatter plot for impulsivity error rate and intra-individual variability in reaction time in Year 6*


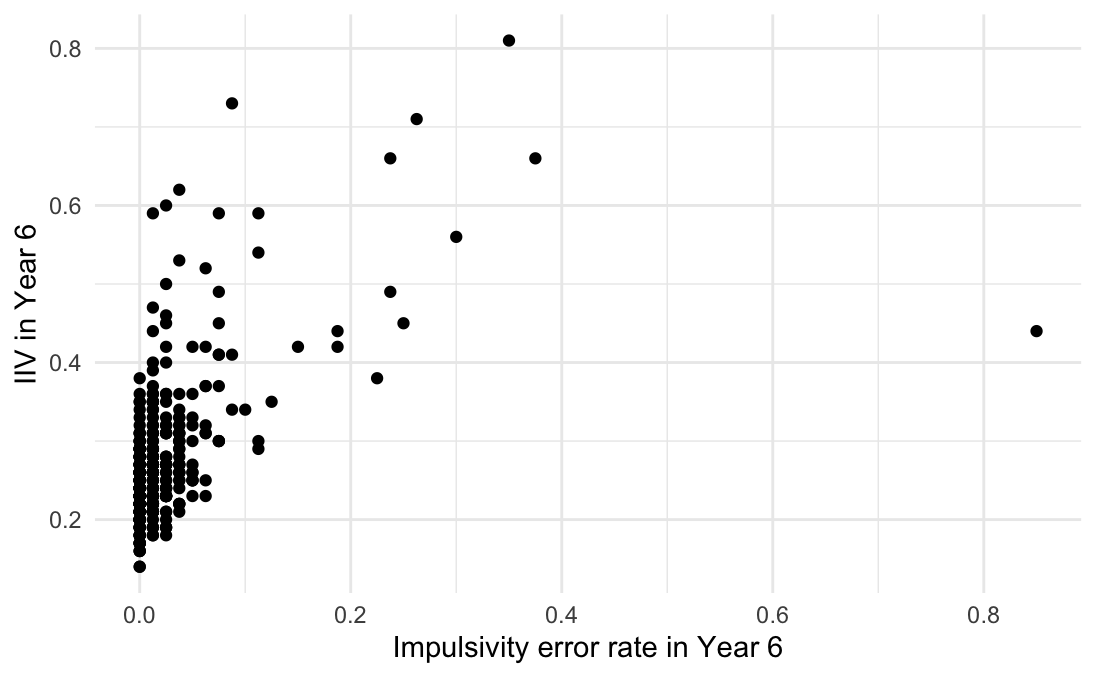


*Note*. IIV = intra-individual variability in reaction time.

**Figure S18**

*Scatter plot for commission error rate and intra-individual variability in reaction time in Year 6*


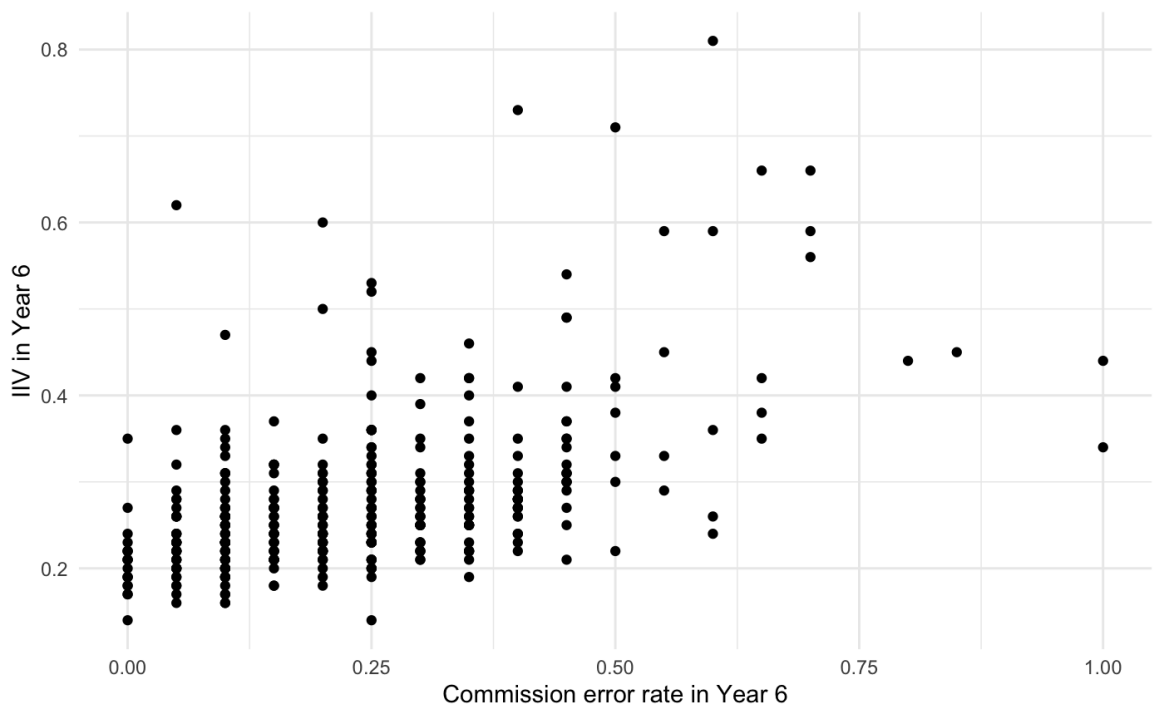


*Note*. IIV = intra-individual variability in reaction time.

**Table S1**

*Comparisons between children who have complete data and those who have missing data in Years 3 and 6 on variables of interest in Year 1*

| Time point | Year 3 | | | Year 6 | | |
| --- | --- | --- | --- | --- | --- | --- |
|  | Complete | Missing | T-test | Complete | Missing | T-test |
| N | 451 | 78 |  | 328 | 201 |  |
| Year 1 variables |  |  |  |  |  |  |
| IWR ^a^ | 10.75% | 10.49% | *t* (94.89) = -.16, *p* = .87 | 10.92% | 10.37% | *t* (427.72) = -.50, *p* = .62 |
| Decoding ^a^ | 20.77% | 19.40% | *t* (97.68) = -.51, *p* = .61 | 20.83% | 20.19% | *t* (425.50) = -.32 *p* = .75 |
| ROWPVT ^a^ | 41.32% | 37.53% | *t* (90.63) = -3.38, *p* = .001 | 41.31% | 39.87% | *t* (399.46) = -2.13, *p* = .03 |
| EOWPVT ^a^ | 39.88% | 36.94% | *t* (86.80) = -2.41, *p* = .02 | 39.88% | 38.79% | *t* (379.40) = -1.48, *p* = .14 |
| Mean RT ^c^ | 592.15 | 599.86 | *t* (78.52) = .60, *p* = .55 | 598.22 | 584.53 | *t* (427.35) = -1.85, *p* = .07 |
| IIV | .35 | .34 | *t* (87.05) = -.60, *p* = .55 | .35 | .34 | *t* (387.19) = -1.41, *p* = .16 |
| Omission ^b^ | 10.36% | 10.33% | *t* (86.71) = -.31, *p* = .98 | 10.67% | 9.81% | *t* (456.60) = -1.22, *p* = .22 |
| Commission ^b^ | 34.40% | 30.91% | *t* (87.05) = -1.43, *p* = .16 | 34.11% | 33.67% | *t* (406.11) = -.26, *p* = .80 |
| Impulsivity ^b^ | 7.50% | 6.16% | *t* (109.00) = -1.27, *p* = .21 | 7.45% | 7.11% | *t* (397.14) = -.37, *p* = .71 |
| PES ^c^ | 125.88 | 163.40 | *t* (67.19) = .99, *p* = .33 | 121.26 | 146.98 | *t* (330.29) = 1.27, *p* = .21 |

*Note*. N= number; ROWPVT = receptive one word picture vocabulary test; EOWPVT = expressive one word picture vocabulary test; RT = reaction time; IIV = intra-individual variability in reaction time; PES = post-error slowing. ^a^ Accuracy rate. ^b^ Error rate. ^c^ in millisecond.

**Table S2**

*Covariance matrices of Go/No-Go measures in Years 1, 3 and 6*

|  | 1 | 2 | 3 | 4 | 5 | 6 |
| --- | --- | --- | --- | --- | --- | --- |
| Year 1 |  |  |  |  |  |  |
| 1. Commission | － |  |  |  |  |  |
| 2. IIV | .45 | － |  |  |  |  |
| 3. Impulsivity | .51 | .62 | － |  |  |  |
| 4. Omission | .05 | .26 | .23 | － |  |  |
| 5. Mean RT | -.12 | .38 | .09 | .34 | － |  |
| 6. PES | -.12 | .18 | -.01 | .09 | .25 | － |
| Year 3 |  |  |  |  |  |  |
| 1. Commission | － |  |  |  |  |  |
| 2. IIV | .45 | － |  |  |  |  |
| 3. Impulsivity | .44 | .52 | － |  |  |  |
| 4. Omission | .22 | .35 | .34 | － |  |  |
| 5. Mean RT | -.08 | .35 | .11 | .34 | － |  |
| 6. PES | -.11 | .07 | -.08 | .00 | .15 | － |
| Year 6 |  |  |  |  |  |  |
| 1. Commission | － |  |  |  |  |  |
| 2. IIV | .51 | － |  |  |  |  |
| 3. Impulsivity | .43 | .50 | － |  |  |  |
| 4. Omission | .26 | .30 | .33 | － |  |  |
| 5. Mean RT | .03 | .11 | .11 | .39 | － |  |
| 6. PES | -.13 | .06 | -.08 | .07 | .09 | － |

*Note*. RT = reaction time; IIV = intra-individual variability in reaction time; PES = post-error slowing

**Exploratory analyses for regular word reading**

As Table S3 and Figure S19 show, children’s regular word reading ability improved over Years 1, 3 and 6. There was a ceiling effect in Year 6.

**Table S3**

*Accuracy rate of regular word reading in Years 1, 3 and 6*

| Time point | Year 1 | | | Year 3 | | | Year 6 | | |
| --- | --- | --- | --- | --- | --- | --- | --- | --- | --- |
|  | N | Mean | SD | N | Mean | SD | N | Mean | SD |
| Regular word reading | 517 | 29.32% | 27.83% | 495 | 70.08% | 25.74% | 377 | 87.86% | 16.11% |

*Note.* N= number; SD = standard deviation.

**Figure S19**

*Developmental trajectories of regular word reading over Years 1, 3 and 6*


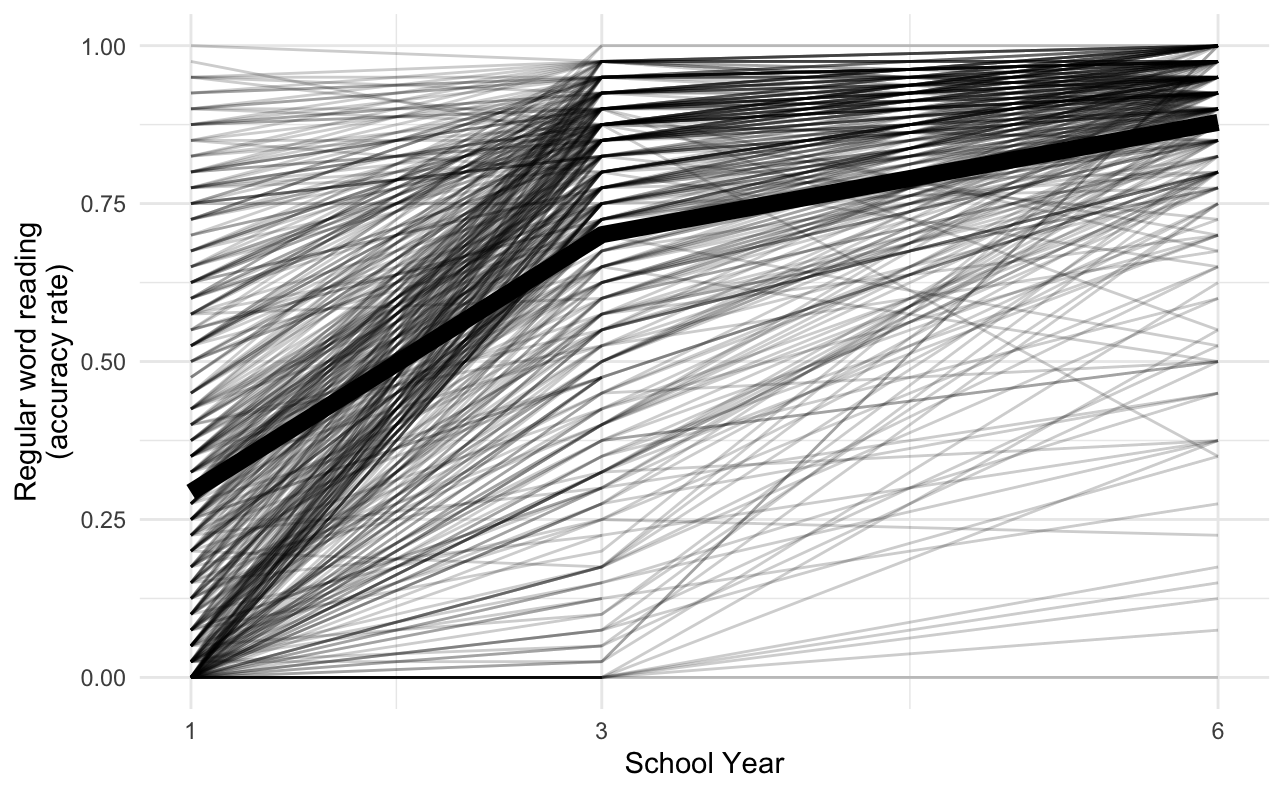


*Note.* The trajectory in bold is based on means at each time point.

**Table S4**

*Correlation matrix for variables of interest (including regular word reading) in Years 1, 3 and 6*

| Variables | 1 | 2 | 3 | 4 | 5 | 6 | 7 | 8 | 9 | 10 | 11 | 12 | 13 | 14 | 15 |
| --- | --- | --- | --- | --- | --- | --- | --- | --- | --- | --- | --- | --- | --- | --- | --- |
| 1. Irregular word reading in Year 1 | － |  |  |  |  |  |  |  |  |  |  |  |  |  |  |
| 2. Regular word reading in Year 1 | .83 | － |  |  |  |  |  |  |  |  |  |  |  |  |  |
| 3. Inhibitory control in Year 1 | -.29 | -.34 | － |  |  |  |  |  |  |  |  |  |  |  |  |
| 4. Decoding in Year 1 | .76 | .89 | -.32 | － |  |  |  |  |  |  |  |  |  |  |  |
| 5. Vocabulary in Year 1 | .55 | .58 | -.39 | .53 | － |  |  |  |  |  |  |  |  |  |  |
| 6. Irregular word reading in Year 3 | .59 | .57 | -.33 | .50 | .56 | － |  |  |  |  |  |  |  |  |  |
| 7. Regular word reading in Year 3 | .51 | .58 | -.33 | .50 | .52 | .83 | － |  |  |  |  |  |  |  |  |
| 8. Inhibitory control in Year 3 | -.27 | -.32 | .56 | -.31 | -.41 | -.36 | -.41 | － |  |  |  |  |  |  |  |
| 9. Decoding in Year 3 | .55 | .63 | -.30 | .57 | .47 | .77 | .89 | -.38 | － |  |  |  |  |  |  |
| 10. Vocabulary in Year 3 | .50 | .51 | -.39 | .45 | .92 | .66 | .62 | -.43 | .57 | － |  |  |  |  |  |
| 11. Irregular word reading in Year 6 | .51 | .52 | -.30 | .45 | .57 | .67 | .66 | -.36 | .61 | .60 | － |  |  |  |  |
| 12. Regular word reading in Year 6 | .36 | .42 | -.31 | .35 | .44 | .60 | .69 | -.38 | .63 | .53 | .80 |  |  |  |  |
| 13. Inhibitory control in Year 6 | -.27 | -.31 | .42 | -.29 | -.42 | -.38 | -.45 | .46 | -.42 | -.50 | -.30 | -.33 | － |  |  |
| 14. Decoding in Year 6 | .40 | .49 | -.31 | .44 | .38 | .61 | .73 | -.37 | .71 | .48 | .73 | .85 | -.32 | － |  |
| 15. Vocabulary in Year 6 | .49 | .49 | -.39 | .44 | .76 | .56 | .52 | -.45 | .48 | .79 | .74 | .59 | -.41 | .55 | － |

**Regression analyses: predicting concurrent regular word reading**

Decoding and vocabulary significantly predicted concurrent regular word reading across time points (ps <.001; effect sizes, .82, .14 in Y1; .78, .19 in Y3; .75, .17 in Y6 respectively).

Adding a regressive path from inhibitory control to regular word reading did not improve fit at any time point. None of the regressive paths from inhibitory control is significant.

**Latent change score models**

***Univariate latent change score model of regular word reading***

A univariate latent change score model (Figure S20) was built for regular word reading, with two latent change scores (circles with ΔRWR inside), one reflecting changes between Year 1 and 3, the other between Year 3 and 6. For the two change scores, each parameter was first constrained to be equal across waves (intercept, yellow arrows; variance, purple arrows; self-feedback parameter, green arrows). Variance parameters were unconstrained, because this significantly improved fit, Δ*X*^2^ (1) = 40.591, *p* < .001. There was less variance in the change score between Year 3 and 6 than between Year 1 and 3. This is in line with the ceiling effect in Year 6. For participants with high accuracy in regular word reading, the change score between Year 3 and 6 was limited by the ceiling effect in Year 6 (the task was too easy for them), and might not be able to reflect their development in regular word reading. Intercept parameters were further unconstrained, because this significantly improved fit, Δ*X*^2^ (1) = 5.958, *p* = .015. The intercept was smaller for the change score between Year 1 and 3 (standardised value 2.200) than that between Year 3 and 6 (standardised value 2.864).

**Figure S20**

*Univariate latent change score model of regular word reading*

*
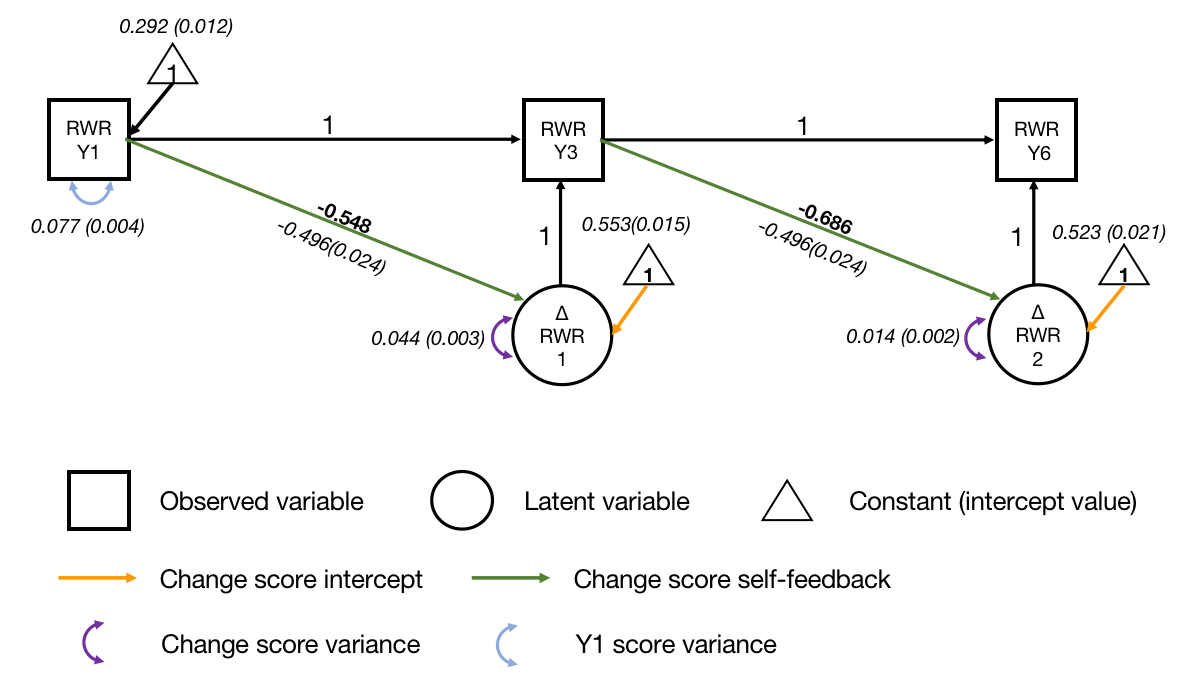
*

*Note*. RWR = regular word reading; Y1 = Year 1; Y3 = Year 3; Y6 = Year 6. Standardised parameter estimates are in roman font. Unstandardised parameter estimates (with standard error estimates in parentheses) are in italics. Key parameters of interest are in boldface.

The final model had a good fit, *X*^2^ (1) = 1.898, *p* = .168, CFI = .998, SRMR = .017, RMSEA = .038, 90% CI = [.000, .122]. Initial regular word reading ability significantly predicted change scores for regular word reading in subsequent years (self-feedback effect; *p*s < .001; *r* = -.548, Year 1 reading predicting the change score between Year 1 and 3; *r* = -.686, Year 3 reading predicting the change score between Year 3 and 6). Participants with better initial regular word reading ability achieved less growth in regular word reading in following years. After accounting for the effect of initial reading ability, there remained a significant amount of variance to be explained in both change scores (*p*s < .001).

***Inhibitory control: no strong evidence for its role in growth in regular word reading***

Inhibitory control variables and two regressive paths were added to the univariate latent change score model, one path from Year 1 inhibitory control to the change score for regular word reading between Year 1 and 3, and the other from Year 3 inhibitory control to the change score between Year 3 and 6 (Figure S21). The regressive parameters were constrained to be equal across waves where possible but were unconstrained if free estimation significantly improved model fit. Covariance was allowed between regular word reading and inhibitory control in Year 1.

**Figure S21**

*Univariate latent change score model of regular word reading predicted by inhibitory control*


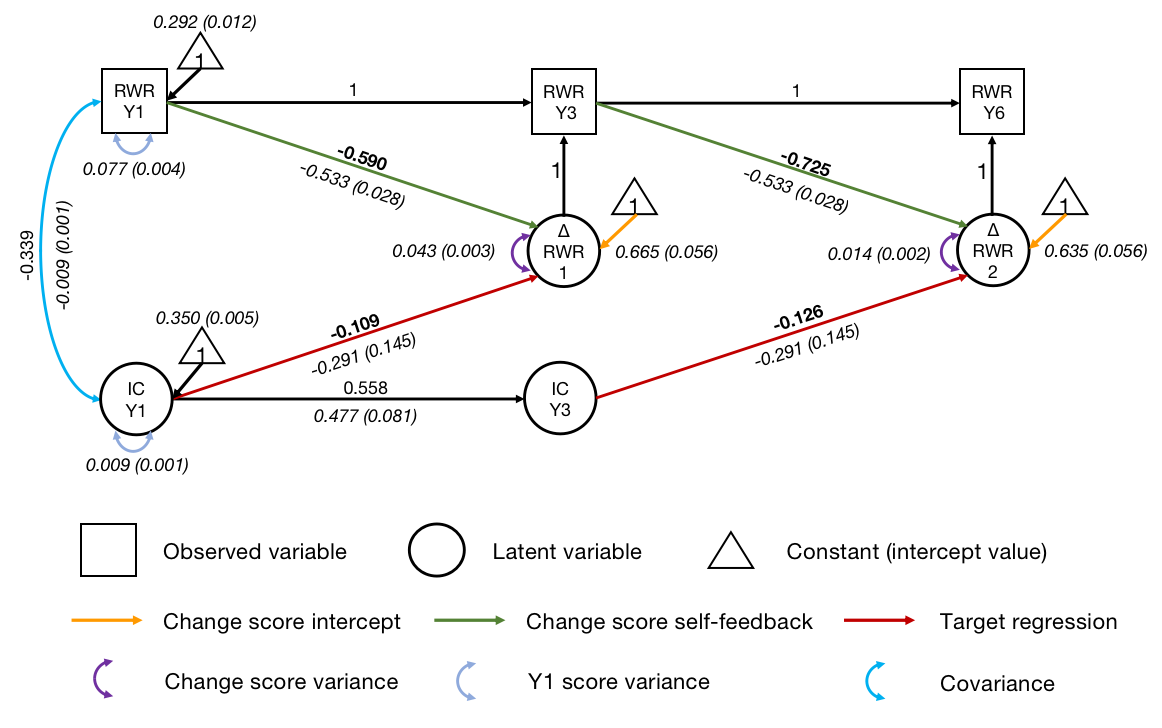


*Note*. RWR = regular word reading; IC = inhibitory control; Y1 = Year 1; Y3 = Year 3; Y6 = Year 6. Standardised parameter estimates are in roman font. Unstandardised parameter estimates (with standard error estimates in parentheses) are in italics. Key parameters of interest are in boldface. Indicators and parameters of inhibitory control variables are not displayed for visual simplicity.

The final model had an acceptable fit, *X*^2^ (22) = 69.585, *p* < .001, CFI = .969, SRMR = .062, RMSEA = .068, 90% CI = [.050, .086]. After controlling for initial regular word reading, both regressive paths from inhibitory control were significant (*p*s = .045). The effects were small (*r* = -.109, from Year 1 inhibitory control to the change score between Year 1 and 3; *r* = -.126, from Year 3 inhibitory control to the change score between Year 3 and 6). Participants with better inhibitory control ability gained more growth in regular word reading. However, fixing the regressive paths to zero did not significantly worsen model fit, Δ*X*^2^ (1) = 3.7706, *p* = .052. There is no strong evidence that inhibitory control predicted growth in regular word reading.

***Decoding: a significant predictor of growth***

Similar analysis steps were applied for decoding, with two regressive paths from decoding (Figure S22). However, model fit was not acceptable, regardless of whether regressive paths were constrained to be equal, unconstrained, or removed. Given that decoding and regular word reading are highly correlated (*r* = .89 in both Year 1 and Year 3), the residual of Year 3 decoding might be correlated to that of the change score for regular word reading between Year 1 and 3. Therefore, their residual covariance was added to the model (as predicted, *r* = .844, *p* < .001). The fit became acceptable, *X*^2^ (4) = 68.114, *p* < .001, CFI = .969, SRMR = .087, RMSEA = .175, 90% CI = [.140, .212]. Regressive paths were then unconstrained, because this significantly improved model fit, Δ*X*^2^ (1) = 11.595, *p* < .001.

**Figure S22**

*Univariate latent change score model of regular word reading predicted by decoding*


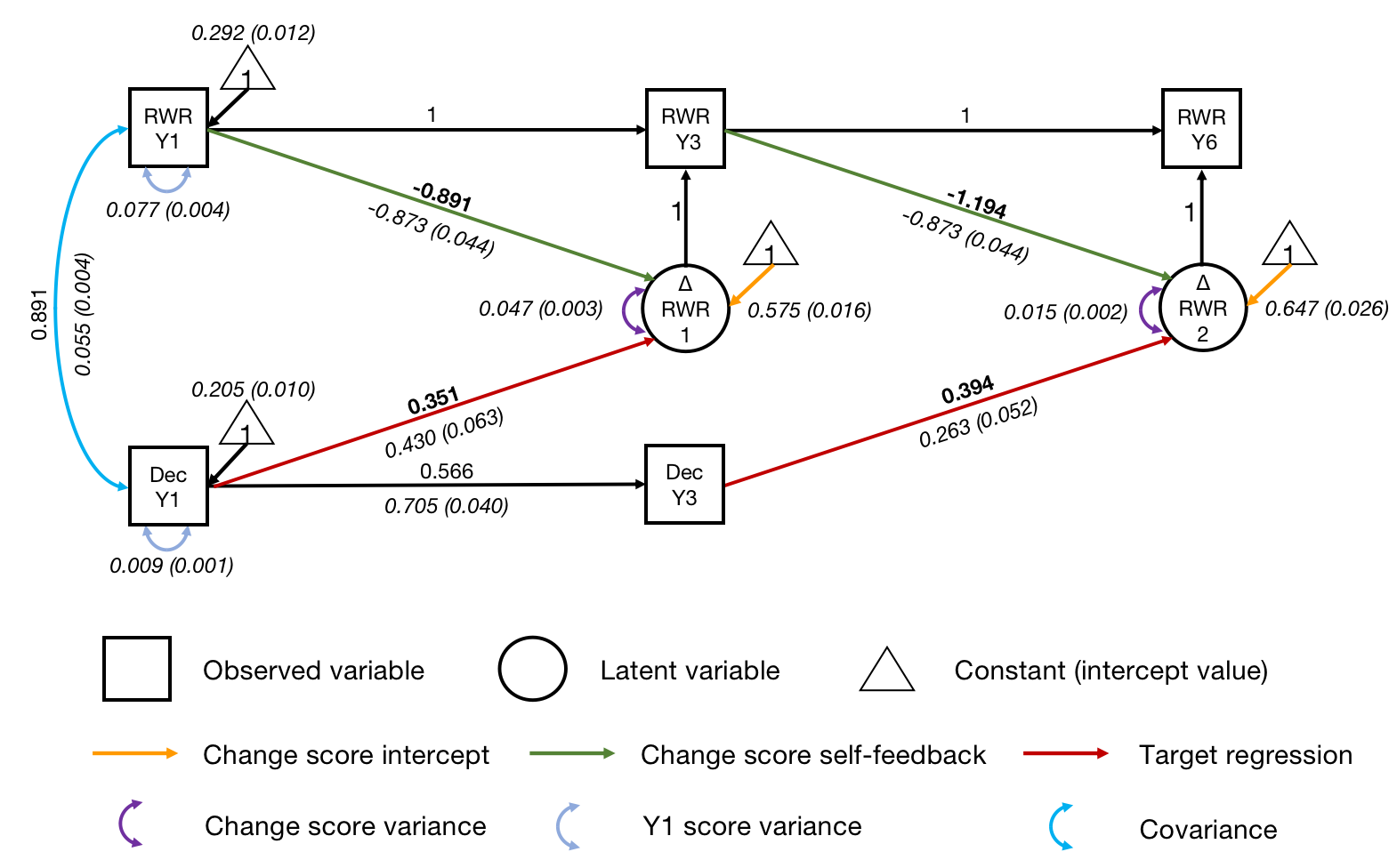


*Note*. RWR = regular word reading; Dec = decoding; Y1 = Year 1; Y3 = Year 3; Y6 = Year 6. Standardised parameter estimates are in roman font. Unstandardised parameter estimates (with standard error estimates in parentheses) are in italics. Key parameters of interest are in boldface. For visual simplicity, the residual covariance between Year 3 decoding and the change score between Year 1 and 3 is not displayed.

The final model had an acceptable fit, *X*^2^ (3) = 88.988, *p* < .001, CFI = .973, SRMR = .044, RMSEA = .187, 90% CI = [.155, .221]. After controlling for initial regular word reading ability, both regressive paths from decoding were significant (*p*s < .001). Participants with better decoding ability achieved more growth in regular word reading. Year 3 decoding had a larger effect size on the growth in regular word reading between Year 3 and 6 (*r* = .394) than Year 1 decoding on the growth between Year 1 and 3 (*r* = .351). Fixing the regressive paths to zero significantly reduced fit, Δ*X*^2^ (2) = 43.65, *p* < .001. This confirms that decoding significantly contributed to growth in regular word reading.

***Vocabulary: a significant predictor of growth***

Similar analysis steps were applied for vocabulary, with two regressive paths from vocabulary (Figure S23). Regressive paths were unconstrained, because this significantly improved model fit, Δ*X*^2^ (1) = 21.18, *p* < .001. The final model had a good fit, *X*^2^ (11) = 63.846, *p* < .001, CFI = .976, SRMR = .034, RMSEA = .093, 90% CI = [.072, .116]. After controlling for initial regular word reading ability, both regressive paths from vocabulary were significant. Participants with better vocabulary knowledge achieved more growth in regular word reading. Year 1 vocabulary had a larger effect size on the growth in regular word reading between Year 1 and 3 (*r* = .374, *p* < .001) than Year 3 vocabulary on the growth between Year 3 and 6 (*r* = .149, *p* = .033). When the regressive path from Year 3 vocabulary was fixed to zero, the model fit significantly reduced, Δ*X*^2^ (1) = 5.178, *p* = 0.022. Fixing both regressive paths to zero also significantly reduced fit, Δ*X*^2^ (2) = 40.642, *p* < .001. Therefore, vocabulary significantly contributed to growth in regular word reading across waves.

**Figure S23**

*Univariate latent change score model of regular word reading predicted by vocabulary*


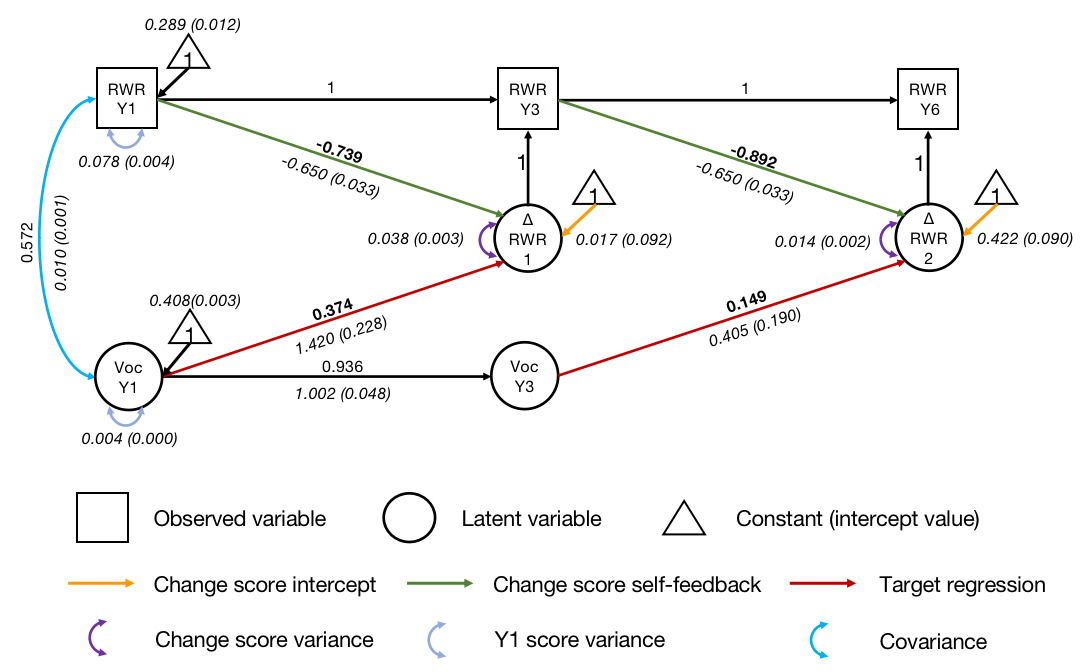


*Note*. RWR = regular word reading; Voc = vocabulary; Y1 = Year 1; Y3 = Year 3; Y6 = Year 6. Standardised parameter estimates are in roman font. Unstandardised parameter estimates (with standard error estimates in parentheses) are in italics. Key parameters of interest are in boldface. For visual simplicity, indicators and parameters of vocabulary variable are not displayed.

***Final model: inhibitory control is not a predictor over and above decoding and vocabulary***

As shown in Figure S24, inhibitory control, decoding, vocabulary, and their regressive paths were added to the univariate latent change score model (regressive paths from Year 1 inhibitory control, decoding, and vocabulary to the latent change score for regular word reading between Year 1 and 3, as well as from Year 3 inhibitory control, decoding, and vocabulary to the latent change score between Year 3 and 6). Covariances were allowed between Year 1 variables. The residual covariance between Year 3 decoding and the change score between Year 1 and 3 was also added to the model.

**Figure S24**

*Univariate latent change score model of regular word reading predicted by decoding and vocabulary*

*
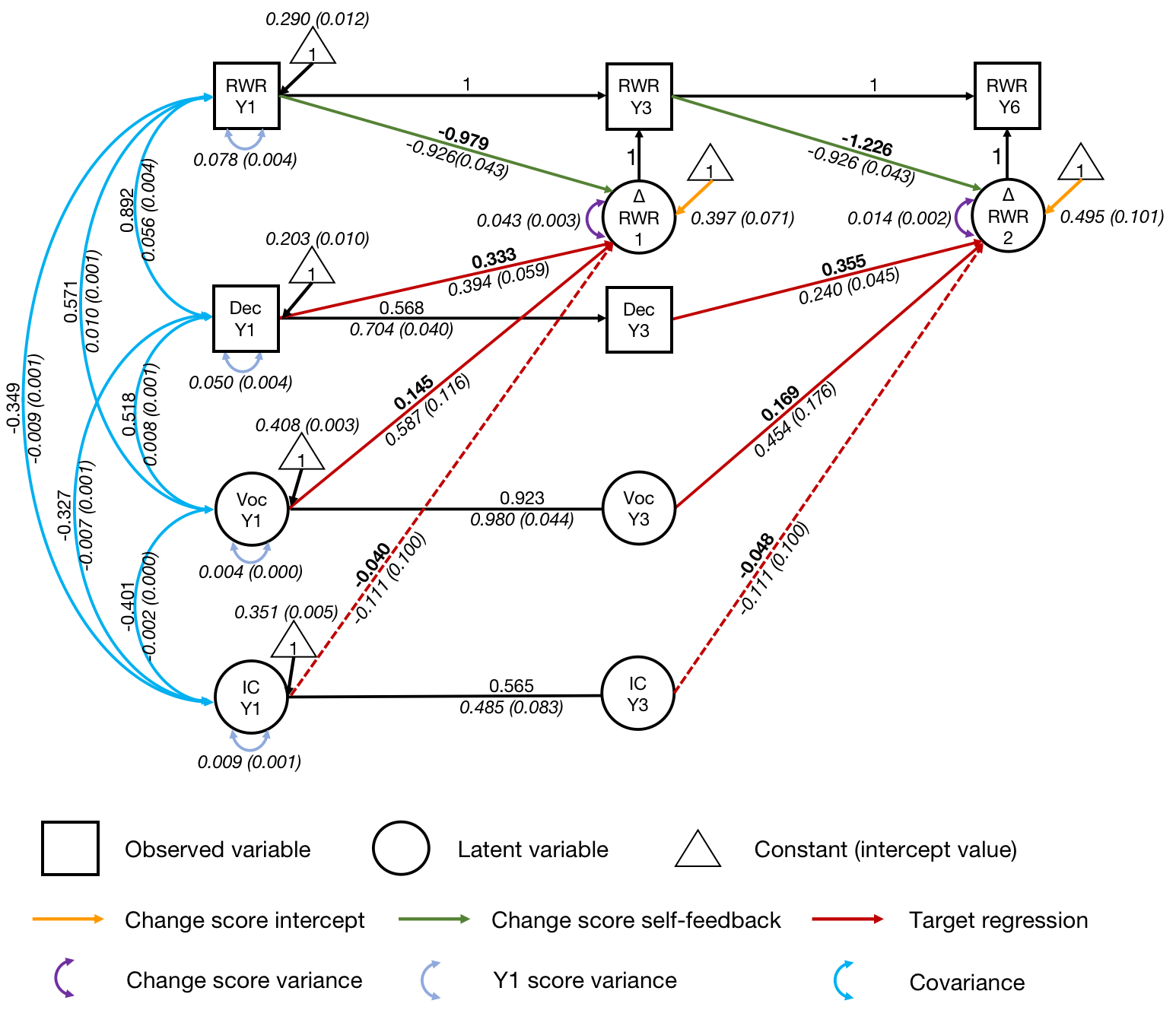
*

*Note*. RWR = regular word reading; Dec = decoding; Voc = vocabulary; IC = inhibitory control; Y1 = Year 1; Y3 = Year 3; Y6 = Year 6. Standardised parameter estimates are in roman font. Unstandardised parameter estimates (with standard error estimates in parentheses) are in italics. Key parameters of interest are in boldface. For visual simplicity, indicators and parameters of vocabulary and inhibitory control latent variables are not displayed. The residual covariance between Year 3 decoding and the change score between Year 1 and 3 is also not displayed.

The model had an acceptable fit, *X*^2^ (75) = 286.930, *p* < .001, CFI = .957, SRMR = .096, RMSEA = .074, 90% CI = [.065, .084]. Regressive paths from decoding were significant (*p*s < .001). The effects were medium (*r* = .333, from Year 1 decoding to the change score between Year 1 and 3; *r* = .355, from Year 3 decoding to the change score between Year 3 and 6). Fixing the regressive paths to zero significantly worsened fit, Δ*X*^2^ (2) = 57.804, *p* < .001. These results suggest that decoding significantly contributed to growth in regular word reading, after accounting for other predictors in the model.

Regressive paths from vocabulary were also significant (*p*s < .001). The effects were small (*r* = .145, from Year 1 vocabulary to the change score between Year 1 and 3; *r* = .169, from Year 3 vocabulary to the change score between Year 3 and 6). Fixing the regressive paths to zero significantly worsened fit, Δ*X*^2^ (2) = 30.218, *p* < .001. Therefore, vocabulary significantly contributed to growth in regular word reading, after controlling for other predictors.

After controlling for initial regular word reading, decoding, and vocabulary, inhibitory control did not significantly predict growth in regular word reading (*p*s = .265). Fixing the regressive paths to zero for inhibitory control did not worsen fit, Δ*X*^2^ (1) = 1.179, *p* = .278. Therefore, inhibitory control did not contribute to growth in regular word reading over and above decoding, vocabulary, and initial regular word reading.
